# Barriers and facilitators to healthy eating in disadvantaged adults living in the UK: a scoping review

**DOI:** 10.1186/s12889-024-19259-2

**Published:** 2024-07-03

**Authors:** Raluca A. Briazu, Fatima Masood, Louise Hunt, Clare Pettinger, Carol Wagstaff, Rachel McCloy

**Affiliations:** 1 School of Psychology and Clinical Language Sciences, University of Reading, Berkshire, UK; 2 Faculty of Health, University of Plymouth, Plymouth, UK; 3 Department of Food and Nutritional Sciences, University of Reading, Reading, UK

**Keywords:** Socioeconomic deprivation, Dietary intake, Environmental barriers, Scoping review, Theoretical domains framework

## Abstract

In the UK people living in disadvantaged communities are less likely than those with higher socio-economic status to have a healthy diet. To address this inequality, it is crucial scientists, practitioners and policy makers understand the factors that hinder and assist healthy food choice in these individuals. In this scoping review, we aimed to identify barriers and facilitators to healthy eating among disadvantaged individuals living in the UK. Additionally, we used the Theoretical Domains Framework (TDF) to synthesise results and provide a guide for the development of theory-informed behaviour change interventions. Five databases were searched, (CINAHL, Embase, MEDLINE, PsycINFO, and Web of Science) for articles assessing healthy dietary intake of disadvantaged adults living in the UK. A total of 50 papers (34 quantitative; 16 qualitative) were included in this review. Across all studies we identified 78 barriers and 49 facilitators found to either impede and/or encourage healthy eating. Both barriers and facilitators were more commonly classified under the Environmental, Context and Resources TDF domain, with 74% of studies assessing at least one factor pertaining to this domain. Results thus indicate that context related factors such as high cost and accessibility of healthy food, rather than personal factors, such as lack of efficiency in healthy lifestyle drive unhealthy eating in disadvantaged individuals in the UK. We discuss how such factors are largely overlooked in current interventions and propose that more effort should be directed towards implementing interventions that specifically target infrastructures rather than individuals.

**Supplementary Information:**

The online version contains supplementary material available at 10.1186/s12889-024-19259-2.

## Introduction

### Background

Healthy diet, namely a diet high in fruit vegetables and legumes and low in sugar, fat and salt [1] is essential to our overall well-being. Poor diet is one the biggest preventable risk factors to ill-health and adherence to healthy eating recommendations has the potential to increase life expectancy and prevent cardiovascular diseases and some cancers [2]. Every year, in the UK, diets that are low in vegetables and legumes are associated with 18,000 premature deaths [3]. In 2018 only 33% of UK adults consumed at least 5 portions of fruit and vegetables a day [4], and less than 1% follow all recommendations set out in the Eat-Well Guide [5, 6]. Worryingly, these numbers are even lower for disadvantaged individuals (herein referred to as those experiencing a state of observable and demonstrable disadvantage relative to the wider society or nation to which an individual belongs to [7]), as diet is subject to vast socioeconomic disparities. Overall, those in the most deprived quintile consume 37% less fruit and vegetables, 54% less fish and 17% less dietary fibre as compared to those in the least deprived quintile [8].

Tackling dietary inequalities is not only beneficial at an individual level but can also improve the social and economic outlook of the country [9], and thus reducing dietary inequalities should be of importance to policy makers in the UK [10]. The Government’s levelling up agenda tried to address longstanding local and regional inequalities by describing its ambition to address poor diet due to its significant contribution to ill health [11]. The Levelling Up programme promised to take forward suggestions from the National Food Strategy [12], a Government commissioned independent review that recommends several ways to reduce diet-related health inequalities. Furthermore, the UK government’s National Productivity Investment Fund has provided crucial funding for research and development projects such as the Transforming UK Food Systems, aimed at transforming the UK food system to benefit the health of people and the environment [13]. As part of this larger project, the Food System Equality (FoodSEqual) project, seeks to provide citizens of disadvantaged communities with choice and agency over the food they consume by co-developing new products, new supply chains and new policy frameworks [14]. However, the UK Government’s current approach to reducing dietary inequalities has received criticism for being biased towards individual responsibility, and for being vague and unambitious [15]. Consequently, calls have been made for the UK government to consider evidence-based interventions which aim to improve diet quality and reduce social inequalities [16]. One important step towards creating interventions aimed at achieving behaviour change such a healthy eating is understanding what determines both the current and desired behaviours.

To do so, there is a need to understand the barriers and facilitators to healthy eating of disadvantaged individuals. Existing reviews of factors that influence healthy eating focus on specific sections of the general populations such as postpartum women e.g. [17], middle aged individuals [18] and those with impaired mobility [19], or focus on countries outside the UK, or are not specific to the UK e.g. [20]. Additionally, most existing reviews do not highlight factors specific to disadvantage. For example, a review of facilitators and barriers to healthy eating in UK young adults referred to individuals with low-income but grouped findings in relation to this group of individuals together with findings regarding ethnicity and collectively referred to these groups as socially excluded [21]. Upcoming systematic reviews specifically propose looking at disadvantaged individuals such as those that are homeless, however again these does not focus on the UK e.g. [22]. This review aims to address this gap in the literature by focusing specifically on disadvantaged adults from the UK. Furthermore, it aims to provide a guide for intervention-targeted policy makers by synthesising findings using the Theoretical Domains Framework (TDF) [23]. The TDF was developed through a collaboration between psychology theorists, health service researchers and health psychologists, by simplifying and integrating a multitude of behaviour change theories with the aim of make theory more accessible and usable across disciplines. In total, the TDF synthesises 33 behaviour change theories and 84 key theoretical constructs related to behavioural change under 14 domains, and thus facilitates the use of psychological theories in the development of interventions. The 14 domains include knowledge; skills; memory, attention and decision processes; behavioural regulation; social/professional role and identity; beliefs about capabilities; optimism; beliefs about consequences; intentions; goals; reinforcement; emotion; environmental context and resources; and social influences.

The framework has been thoroughly validated [23] and used to explain implementation problems, develop theory-informed behaviour change interventions, and assess which theoretical domains are relevant to particular interventions e.g. [24–26]. Furthermore, the TDF links seamlessly to the Behavioural Change Wheel (BCW) [27], a framework designed to aid intervention designers in moving from a behavioural analysis to an evidence-based intervention method. The framework provides a link between theorised sources of behaviour, intervention functions and policy categories [28]. Therefore, by using the TDF to summarise barriers and facilitators to healthy eating in UK disadvantaged communities we aim to identify whether intervention designers need to focus on specific domains in order to inform future interventions within this context.

### Purpose of the review

In this scoping review, we aimed to summarise and systematically chart the available empirical evidence regarding the barriers and facilitators to healthy diet encountered by disadvantaged UK adult. The overarching review question was: *What is known about the factors that encourage or impede the healthy diet of disadvantaged adults living in the UK?* In addressing our overarching research question, our specific objectives were to: (1) identify the barriers and facilitators to healthy diet from the perspective of disadvantaged UK adults using the theoretical lens of the TDF; and (2) help inform the future development of theory led behavioural change interventions in this target population.

## Method

We conducted a mixed-method scoping review of published peer-reviewed primary research. Scoping reviews provide an overview of available literature on a topic, they examine the extent, type, range and nature of evidence in order to understand the current status of the knowledge related to a topic of interest, without assessing the quality of studies included [29–32]. This approach is recommended when aiming to summarise a body of work that is diverse in its methodology or discipline [30, 31].

### Review protocol

We used the framework proposed by Levac and colleagues [30] alongside the Population, Concept, and Context (PCC) framework from the Joanna Briggs Institute [33]. The review was reported in accordance with the PRISMA Extension for Scoping Reviews [34] (PRISMA-ScR; see Appendix A). An initial review protocol was devised and agreed by the research team in September 2021 (available from authors on request). As advised by Levac and colleagues [30] we adopted an iterative approach to the review process, engaging with each stage in a reflexive way, repeating steps where necessary to ensure comprehensiveness, therefore we updated the protocol in May 2022 to accommodate revised study selection criteria.

### Eligibility criteria

Studies of any design which qualitatively and/or quantitatively examined factors influencing healthy eating in disadvantaged communities or individuals in the UK were eligible for inclusion. We aimed for review papers to be included as long as they addressed the research question^1^.

Initially, we did not include age as an exclusion criteria, however once familiarity with the literature was gained, studies focused on children or adolescents were found to differ in scope in comparison to the other articles. Hence, inclusion criteria were revised to include only studies of individuals aged 18 and over. Studies of young adults that also included participants aged 16- or 17-year-old, were included if most participants were aged over 18. Studies that included adult participants reporting on children’s dietary intake were excluded.

Our assessment of healthy eating was guided by the World Health Organisation’s [1] definition of healthy eating, namely a diverse diet rich in fruit and vegetables and legumes, that limits intake of saturated fat, free sugars and salt. We included studies specifically assessing food intake and food purchases. We excluded studies that looked at intended consumption or perceptions of healthy eating. We also excluded studies that assessed food insecurity without including specific healthy eating indicators, because whilst food insecurity is strongly associated with a poor diet the concept of food insecurity is multi-dimensional and includes concepts that are different to healthy eating such as feeling unsatisfied [35].

In terms of barriers and facilitators, factors enhancing or positively influencing healthy eating were regarded as facilitators, whilst factors impeding healthy eating were defined as barriers. In both cases we only considered physical, psychological or socio-ecological factors. Biological and genetic factors such assigned sex were not included. Furthermore, socio-demographic characteristics such as age and nationality were also excluded. Studies were only included if a clear link could be drawn between all variables of interest, namely barriers or facilitators, healthy eating and disadvantage.

We included studies that measured socio-economic status in a standardised way such as Indexes of multiple deprivation (IMD) (e.g. English IMD, Welsh IMD or Scottish IMD) [36] or included food bank attendees, homeless or unemployed individuals as these are individuals known to experience the highest level of disadvantage [37, 38]. Studies using low household income as an indicator of SES were included, as low-income is the highest risk factor for disadvantage [39]. Studies using social class indicators based on occupation were excluded unless results were specific to social class E, representative of those who are unemployed and have the lowest grade occupations [40]. Studies that only used single proxies for disadvantage such as educational level or general occupation (unless specific to social class E) were excluded. Studies that compared disadvantaged individuals or communities against other types of communities or groups of individuals were included as long as results pertaining to the disadvantaged communities or individuals could easily be extracted.

We only included studies that focused on a UK population or individuals from UK countries, namely England, Wales, Scotland and Northern Ireland. Studies that also included groups of participants from outside the UK were only included if results pertaining to UK disadvantaged communities or individuals could be easily extracted.

### Literature search

A search of the following electronic databases was conducted: the Cumulative Index to Nursing and Allied Health Literature–CINAHL (EBSCO), Embase (Ovid), MEDLINE (Ovid), PsycINFO (EBSCO), and Web of Science. The search was guided by a more general research aim, namely investigating the diet intake of disadvantaged communities or individuals in the UK. An experienced information specialist at the University of Plymouth was consulted prior to starting the search to develop the search strategy to ensure the review process followed a systematic approach. The search strategy was the same as the one used in the scoping review by Hunt and colleagues [41]. Significant terms derived from the main research question were selected and expanded to create a comprehensive list of primary search terms and variants. Population search terms related to individuals of all ages were included. Context related search terms comprised terms such as ‘low-income’, ‘poverty’, ‘deprivation’ and ‘vulnerable populations’. Concept related search terms referred to diet in general such as ‘diet’, ‘healthy diet’ ‘nutritious’ and ’food quality’ as well as means of dietary intake such as ’eat’ ‘consume’ and ‘family meal’. The search also included terms related to the UK, including one for each UK nation. Searches for population, context, concept and location terms were combined using OR, the subsequent results were combined with the location results using AND. Finally, the research was limited to research published between 01.01.2010 and 29.09.2021, in English language, using human participants. Search strategies for each database can be found in Appendix B. We also performed manual searches using the reference lists of articles that met inclusion criteria.

### Data charting

Following the search, all identified citations were collated and uploaded into a Microsoft Excel [42] spreadsheet and duplicates were removed. Initially, titles and abstracts were screened for assessment against the inclusion criteria for the review. The full text of selected citations was assessed in detail against the inclusion criteria by two independent reviewers. Reviewers were blinded to journal or author information. Reasons for excluding sources of evidence at full text were recorded. Disagreements that arouse between the reviewers at each stage of the selection process were resolved through discussion, or by consulting a third reviewer.

The following information was extracted: (1) year of publication, (2) study design, (3) research design, (4) socio-economic indicator used to identify disadvantage, (5) dietary variables assessed by the study, (6) healthy eating indicator assessment method, (7) barriers, (8) facilitators, (9) results pertaining to barriers and facilitators. For intervention studies, the intervention itself was classed as a facilitator if this positively influenced healthy eating. For qualitative studies, barriers and facilitators were extracted if they were mentioned as part of a theme or subtheme in the discussion section.

### Synthesis of results

After initial extraction, the Theoretical Domains Framework was used to categorise the barriers and facilitators. Two reviewers independently categorised the extracted barriers and facilitators into the 14 TDF domains based on a coding manual with theoretical definitions for each domain [22]. Two reviewers initially reached a 70% agreement on the categorisation of barriers and facilitators. Discrepancies occurred due to the overlap between domains. It has been acknowledged that TDF domains are not mutually exclusive [43]. For example, smoking could be considered a decision or a habit and thus linked to identity or alternatively to the context one lives in. The reviewers solved discrepancies through discussion, rereading source material, and collaboration. When no agreement could be reached, the opinion of a third reviewer determined the final result. Final TDF categorisations were reviewed and discussed with the entire team if any alternative categorisations were plausible.

## Results

**Fig. 1:**
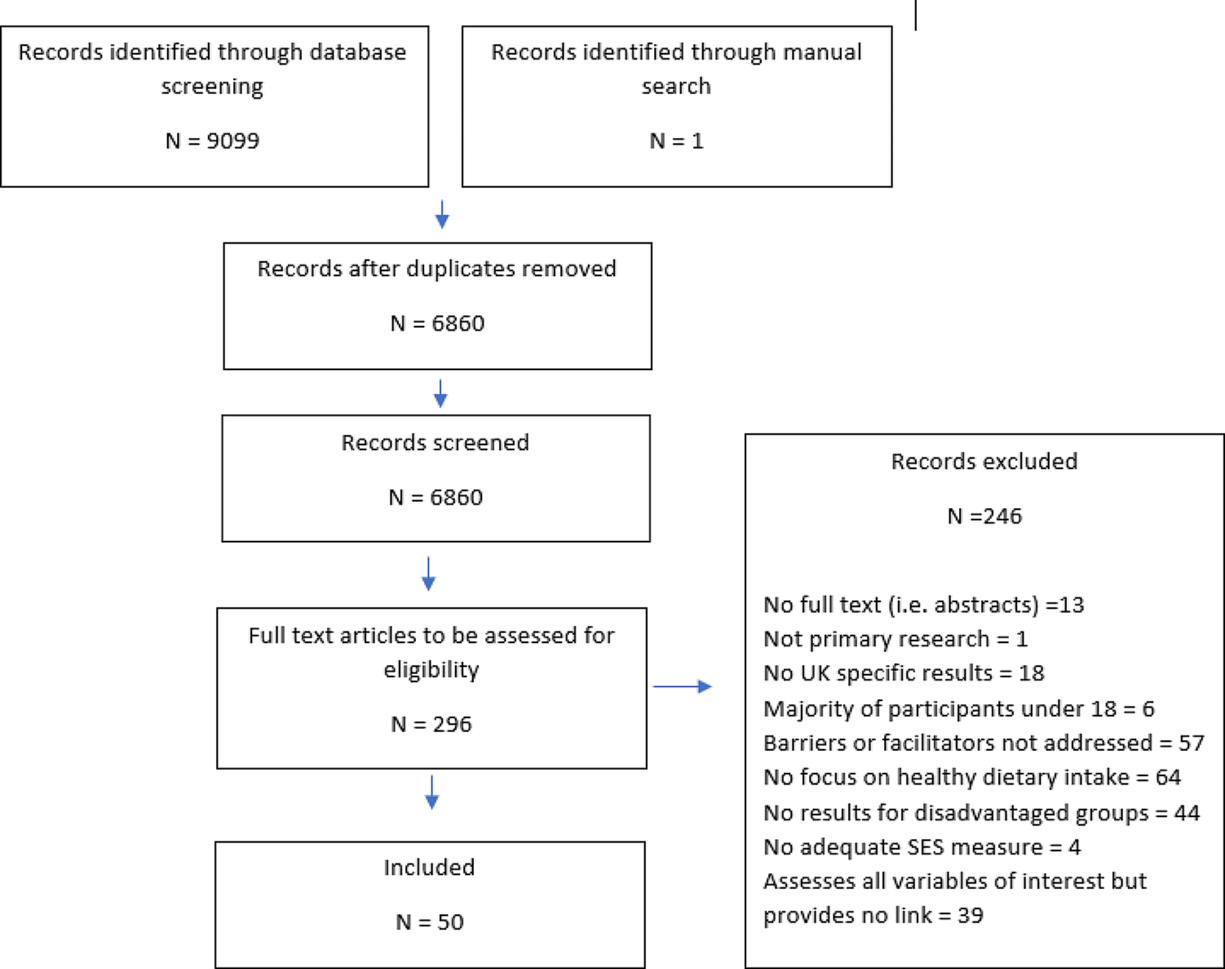
Flowchart of study selection process

Overall, 9099 records were identified through database searches, and one was identified through the manual search, the abstracts of 6860 articles were screened for eligibility of which 6564 were excluded. Of the remaining 296 papers that were included in the full-text screen, 246 were excluded as they did not meet eligibility criteria (see Appendix C for a list of all excluded records), leaving 50 studies for inclusion. Figure 1 shows the study selection process.

Table 1 summarizes the characteristics of studies included in this review. The reviewed articles included 42 studies that found factors that were either positively or negatively associated with healthy eating and 8 studies that included non-significant findings. Most studies, 68% (*n* = 34) employed quantitative methods, and the remainder used qualitative methods (*n* = 16). Studies that used a mixed methodology were classified as either qualitative or quantitative depending on the method which was used to gather data included in this review. Only 14 studies included participants from across the UK, with the most being conducted in England (*n* = 26). A third of studies (32%) focused on variables related to general healthy eating indicators (i.e. healthy eating, diet quality, diet variety), whilst the majority focused on specific indicators that could be linked to healthy eating such as fast-food, sugar or processed meat consumption. Fruit and vegetable consumption was the most studied single indicator of healthy eating and was assessed by 47% of studies included in this review.

**Table 1:** Characteristics for each study included in the review

First author	Type of data	Country	*N*	Disadvantage Indicator	Healthy Eating Indicator	Assessment method	Barriers	Facilitators
Baird et al. [44]	Quantitative	England	527	Living in disadvantaged area	Diet quality	20-item FFQ^*^	Y	N
Barker et al. [45]	Qualitative	England	112	Food bank users	Food quality and variety	Multiple-pass 24-hour recall	Y	N
Barton et al. [46]	Qualitative	Northen Ireland	42	Northern Ireland Multiple Deprivation Measure	Healthy eating	Qualitative interviews	Y	N
Bellis et al. [47]	Quantitative	UK	7047	the English Index of Multiple Deprivation 2011 (IMD) and the Welsh Index of Multiple Deprivation 2014 (WIMD)	Fruit and vegetable consumption	Single item questionnaire	Y	Y
Blow et al. [48]	Qualitative	England	13	Low-socioeconomic location	Take-away consumption	Semi-structured interviews	Y	Y
Burgoine et al. [49]	Quantitative	England	51,361	Household income	Consumption of processed meat	Dietary screener FFQ	Y	N
Davison et al. [50]	Qualitative	Northen Ireland	14	Not in education, employment or training	Healthy eating	Focus groups & Semi-structured interviews	Y	Y
Davison et al. [51]	Quantitative	Northen Ireland	168	Not in education, employment or training	Healthy diet Junk food and fast-food choice	19-item FFQ	Y	Y
Ejlerskov et al. [52]	Quantitative	UK	unspecified	Household social grade E	Purchase of unhealthy checkouts foods	Sells data	N	Y
Fielden et al. [53]	Quantitative	England	85	Low socioeconomic status	Fruit and vegetable Consumption	24 h recall method	N	Y
Forde & Solomon-Moore [54]	Qualitative	England	8	Food bank users	Sugar consumption	Semi-structure interviews	Y	N
French & McKillop [55]	Quantitative	Northen Ireland	499	Household income	Fruit and vegetable consumption Fast-food consumption	Single item questionnaire for each variable	Y	N
Garcia et al. [56]	Quantitative	Scotland	102	Living in disadvantaged area	Ready meal consumption Fruit and vegetable consumption Ready meal consumption	3-item FFQ	N	Y
Gardner et al. [57]	Quantitative	England	4418	Living in disadvantaged area	Fried snack consumption Fruit and vegetable consumption	7-day recall for each variable	N	Y
Garthwaite et al. [58]	Qualitative	England	42	Food bank users	Healthy diet	Semi-structured interviews	Y	N
Goodall et al. [59]	Quantitative	England	114	Living in disadvantaged area	Fruit and vegetable consumption	125-item FFQ & 3-day food diary	N	Y
Griffith et al. [60]	Quantitative	UK	6235	Household income	Nutrient composition of households’ shopping baskets	Purchase data	N	Y
Hillier et al. [61]	Quantitative	England	128	Living in disadvantaged area	Percentage of food energy from fat (%fat) Number of portions of fruit and vegetables (FV)	Computerised previous day recall programme	N	Y
Holmes & Roberts [62]	Quantitative	UK	662	Index of material deprivation	Diet quality	24-h recall multi-pass method recorded on 4 consecutive days	Y	Y
Hutchinson et al. [63]	Quantitative	England	462	Living in disadvantaged area	Vegetable intake Snack consumption	Single item FFQ for each variable	N	Y
Jenning et al. [64]	Quantitative	England	255	Living in disadvantaged area	Fruit and vegetable intake	2-item FFQ	N	Y
Jones et al. [65]	Quantitative	Wales	151	Welsh Index of Multiple of Deprivation	Improvement in healthy diet	Single item questionnaire	N	Y
Kearns & Mason [66]	Quantitative	Scotland	1283	Living in disadvantaged area	Fruit and vegetables consumption Sweet snacks consumption Frequency of fast-food and home-cooked main meals consumption	4-item FFQ	Y	Y
Lucas et al. [67]	Qualitative	England	107	Household income	Fruit and vegetable consumption	In-depth interviews	N	Y
Mackenbach et al. [68]	Quantitative	England	10,020	Household income	Fruit and vegetable consumption	130-item, semi-quantitative FFQ	Y	N
Marty et al. [69]	Quantitative	UK	1743	Household income	Total energy of food chosen to consume	Virtual food purchasing	N	Y
McFadden et al. [70]	Qualitative	England	85	Household income	Fruit and vegetable purchasing Healthy diet	Focus groups and qualitative participatory workshops	N	Y
Ntouva et al. [71]	Quantitative	UK	2796	Household income	Non-milk extrinsic sugars consumption	24-hour recall ‘multiple pass’ method	Y	Y
Ohly et al. [72]	Qualitative	England	11	Low-income	Healthy eating	Semi-structured interview	N	Y
Packard et al. [73]	Quantitative	Scotland	666	Scotland Index of Multiple Deprivation	Fruit and vegetable consumption	21-item FFQ	N	Y
Paudyal et al. [74]	Qualitative	Scotland	28	Homeless individuals	Healthy eating	Semi-structured interview	Y	Y
Pechey et al. [75]	Quantitative	UK	1509	Multiple indices of material deprivation	Pre-packaged snack food	Participant choice	N	Y
Petchey et al. [76]	Quantitative	UK	732	Household income	Fresh fruit, cheese and cake consumption	Single item FFQ for each variable	Y	N
Phillips et al. [77]	Quantitative	England	3986	English Indices of Multiple Deprivation	Unhealthy eating	FFQ	N	Y
Power et al. [78]	Qualitative	England	22	Household income	Healthy eating	Focus groups	Y	N
Puddephatt et al. [79]	Qualitative	England	24	Food bank users	Healthy food choices	Semi-structured interviews	Y	N
Renton et al. [80]	Quantitative	England	4107	English Indices of Multiple Deprivation	Fruit and vegetable consumption	FFQ	N	Y
Russell et al. [81]	Quantitative	England	11,243	Index of Multiple Deprivation (IMD)	Fruit and vegetable consumption	Single item FFQ	Y	N
Scantlebury et al. [82]	Quantitative	UK	64,874	Benefits receivers	Fruit and vegetable consumption	24 h recall	N	Y
Sprake et al. [83]^†^	Qualitative	England	24	Food bank users	Healthy eating	Semi-structured interviews	Y	Y
St Clair et al. [84]	Qualitative	England	unspecified	Living in disadvantaged area	Fruit and vegetable consumption	Semi-structure interviews & group interviews	N	Y
Stead et al. [85]	Quantitative	UK	53,367	Living in disadvantaged area	Healthy meal deal purchase Low fat milk purchasing	Electronic point of sale data	N	Y
Stevens et al. [86]	Quantitative	UK	228	Household income	Healthy eating	MCQ FFQ Optional 24 h recall test	Y	N
Thompson et al. [87]	Qualitative	England	26	Living in disadvantaged area	Healthy shopping	Go-along interviews	N	Y
Thompson et al. [88]	Qualitative	England	14	Food bank users	Adequate nutrition	Semi-structured interview	Y	N
Thornton et al. [88]	Quantitative	UK	3738	Multiple indices of material deprivation	Fruit and vegetable consumption Low fat milk consumption	Multiple FFQs	Y	Y
Tong et al. [89]	Quantitative	UK	12,417	Household income	Adherence to Mediterranean diet	130-item semi-quantitative FFQ	Y	N
Tsakos et al. [90]	Quantitative	UK	3728	Household income	Fruit and vegetable consumption		Y	N
Vogel et al. [91]	Quantitative	England	60	Index of Multiple Deprivation (IMD) deciles	Fruit and vegetable purchasing Confectionary purchasing Diet quality	20-item FFQ & 2-item FFQ	N	Y
Watts et al. [92]	Quantitative	England	1120	English Indices of Multiple Deprivation	Fruit and vegetable consumption	Validated FFQ	Y	N

* FFQ = food frequency questionnaire

^†^ This study used a mixed methods approach, however the results used in this scoping review were based only on the qualitative findings

Across all studies we identified 78 barriers and 49 facilitators found to either impede and/or correspondingly encourage healthy eating. We further identified 4 barriers and 7 facilitators found to have no impact on healthy eating in disadvantaged communities.

Tables 2 and 3 summarize all the barriers and facilitators organized by data type (quantitative and qualitative) and TDF categorization. Below we provide a summary of findings by TDF domain, in addition to the table we also summarize the few studies that focused on interventions aimed at several factors. In the summary below, we include an example study for each barrier and facilitator, for the full list please refer to Tables 2 and 3.

**Table 2:** Barriers to healthy eating for each TDF domain

TDF domain	Quantitative	Qualitative
Knowledge	Low educational attainment [44, 92]	Lack of knowledge about nutritional value of food [74]
	Leaving school at 16 [51, 71]	Limited knowledge about sugar [54]
Skills		Cooking skills vs. variety [48]
		Lack of skills to prepare food [74]
Social/ professional role identity		Routine and traditions [48]
Beliefs and capabilities	Low sense of control [44] Low levels of self-efficacy [44]	Perceived lack of control [50]
Optimism	None identified	None identified
Beliefs about consequences	None identified	None identified
Reinforcement	None identified	None identified
Intentions		Motivation (affected by personal circumstances in relation to financial stability) [54]
		Shopping to satisfy hunger [74] Eating to survive [83]
Goals		Perceived need to create more time for leisurely activities [46]
Memory, attention and decision processes	None identified	None identified
Environmental context and resources	Financial hardship [55] Smoking [62] Unemployment [92] High cost of healthy food [68, 86, 89] Presence of children in the household [88] Single-parent household [66] Good internal home quality [66] Adverse childhood Experience [47] Childhood Violence [81] * Lack of time [86] Poor appetite [62] Difficulty in chewing [62] Poor dental status [90] High -proportion of fast-food outlets [49] Inability to work [92] Long-standing illness [66] Anti-social behaviour in neighbourhood [66] Eating dinner on lap rather than at Table [62]	Issues related to financial hardship [45, 48, 74, 78, 83, 87]
		High cost of fresh food [46, 58, 78, 79] Low cost of take-away food [46, 50] Transport issues [43, 78] Poor nutritional value of donated food [93] Lack of flavour due to out of season production [46] Lacking or saving time [48] Takeaway availability [48, 50] Lack of facilities to store, cook or warm food [74] Accessibility of shops [78, 79] Physical health concerns [54, 74, 80] Poor access to healthy meals [54, 74] Upbringing [54] Reliance on processed foods [54]
Social influences	Eating alone [62]	Bonding with others [48] Being part of a community [48] Influential others [48] Cultural acceptance of sugar consumption [54]
Emotion	Fatigue [86] Feeling stressed [86] Low adult well-being * [81]	Lack of psychological well-being [46] Mental Health concerns [54] Emotional state (need for emotional boost) [54] Worsened health outcomes [80]
Behavioural regulation		Lack of efficacy to engage in a healthy lifestyle [50] Food rationing strategies [79]

* Indicate studies that found the factor not to significantly impede healthy eating or to reduce inequalities

**Table 3:** Facilitators to healthy eating for each TDF domain

Domain	Quantitative	Qualitative
Knowledge	Finishing education at 18 [71] High education [66] Health information leaflets [59] Energy labelling in fast food restaurant [69]*	Mid-high education [88] Adequate knowledge on the importance of healthy meals [74]
Skills	Cooking skills programme [30, 56] *	
Social/ professional role and identity	High extraversion [73] High sense of coherence [73]	Being employed or in education [88]
Beliefs about capabilities	High food self-efficacy [51] High general self-efficacy [73] High degree of self-esteem [73] Self-affirmation [53]	Personal agency [50]
Optimism	Low hopelessness [73]	None identified
Beliefs about consequences	None identified	None identified
Reinforcement	None identified	None identified
Intentions	None identified	None identified
Goals	Pledge on dietary intake [61] *	None identified
Memory, attention and decision processes	High Food involvement [51] Implicit liking of fruit [76] * Perception of fruit (i.e. perceived healthiness, satiety and value for money) [76] *	Personal values relating to food choice [48]
Environmental context and resources	Always available adults [47] Childhood happiness [81] * Creative activity participation [80] Cultural event attendance [80] Eating at the Table [62] Good appetite [62] Not smoking [62] Product placement in shops [91] Increasing availability healthier options * [69, 75] * Community engagement programme [77] Mobile food store [64] Clear check-out policies [52] Good internal home quality [66] Anti-social behaviour in neighbourhood [66] Good local services and amenities [66] Long standing illness [66] Two—parent households [66]	Healthy Start vouchers [60, 67, 70, 72, 82] * Charitable meals [83] Increasing access through community gardening [89*]
Social influences	Support from lay community trainers [59] *	Family and friends support [74] Positive role of healthcare professional advice [74] Proxy agency [50] Collective agency [50]
Emotion	None identified	None identified
Behavioural regulation		Damage control [48] Restricted and budgeted shopping style [87]

* Indicate studies that found the factor not to significantly facilitate healthy eating or to reduce inequalities

### Knowledge

Low educational attainment was identified as a barrier by four different quantitative studies e.g. [43] (see all studies in Table 1) and conversely higher levels of education were identified as a facilitator e.g. [66]. Limited knowledge regarding foods such as nutritional value [74] or knowledge about sugar [54] were also identified as a barrier. Having adequate knowledge of healthy meals was also found to facilitate healthy eating [74]. However, Marty et al. [69] found that providing calorie labelling on menus, an approach aimed to encourage healthy food consumption based on increasing nutritional knowledge, did not significantly encourage healthier consumption in participants with a low-socio economic position.

### Skills

Low cooking and food preparation skills were found to be a barrier e.g. [74]. Blow and colleagues [48], specifically highlighted that individuals often feel their cooking skills cannot match their requirement for variety in diet thus leading to the purchase of take-away meals. Accordingly, two studies found that programmes focused on increasing cooking skills can facilitate healthy eating e.g. [56]. However, Hutchinson et al. [63] found that attending a cooking skill programme could increase consumption of fruit and vegetables and decrease consumption of snacks irrespective of whether individuals were disadvantaged or not.

### Social professional role and identity

One study found that following routines and traditions could encourage unhealthy eating [48]. Being employed or in education [88], and personal factors such as extraversion and high sense of coherence emerged as facilitators [73].

### Beliefs about capabilities

Perceived lack of control over one’s life [44], and over food [50] was identified as a barrier. This was mirrored in the facilitators, as having personal agency was identified as enabling healthy eating [450]. Similarly low-self efficacy was identified as a barrier [44] whereas high food self-efficacy was identified as a facilitator by a separate study [51]. Additionally, general high self-efficacy was also found to predict higher fruit and vegetable intake [73]. Self-affirmation and a high degree of self-esteem were also found to facilitate healthy food choices [53].

### Optimism

No barriers were identified within this domain, but a low level of hopelessness was found to predict a higher consumption of fruit and vegetables [73].

### Beliefs and consequences

No barriers or facilitators were identified in this domain.

### Reinforcement

No barriers or facilitators were identified in this domain.

### Intentions

No facilitators were identified in this domain, however three qualitative studies identified three different barriers. First, low motivation, which was affected by personal circumstances in relation to financial stability was found to affect efforts to reduce sugar consumption [54]. Additionally, in two studies healthy food options were disregarded due to the need to satisfy hunger or to survive e.g. [83].

### Goals

No significant facilitators were identified in this domain, however the perceived need to create more time for leisurely activities was identified as a barrier [46]. Interestingly, Hellier and colleagues (2012) [61] found that setting healthy eating relating goals did not improve dietary intake when compared to standard advice-giving techniques.

### Memory, attention and decision processes

No barriers were identified in this domain. For this domain one study identified high food involvement, namely the level of importance of food in a person’s life, as a facilitator [51]. Personal values relating to food choice were identified as a facilitator [48]. Implicit liking of fruit and perception of fruit (i.e. perceived healthiness, satiety and value for money) was shown not to significantly explain the relationship between socio-economic status and frequency of fruit consumption [76].

### Environmental context and resources

Most factors were identified as being part of this domain. Most studies (74%) included at least one factor that could be classified as either a barrier or a facilitator in this domain. Within this domain, by far the most common factor was related to financial issues such as unemployment, inability to work, financial hardship and cost of food, identified by 19 separate studies e.g. [45, 93]. More specifically, high cost of healthy food e.g. [78, 86] and low cost of unhealthy foods [50] were identified as a barriers whereas charitable meals [83] and interventions that targeted cost such as healthy start vouchers enabled healthier diets e.g. [70]. However, although healthy start vouchers were identified as a facilitator by three studies, one quantitative longitudinal study [82] found that Healthy Start eligible families did not increase their fruit and vegetable intake more than other families following the introduction of Healthy Start in 2006 and up to 2014.

General ill health but also dental health that could lead to difficulty in chewing, particularly in men were also identified as barriers e.g. [62, 66]. One study specifically identified smoking and poor appetite in elderly women as a barrier [62]. Conversely, the same study found good appetite and not smoking to be associated with a higher intake of fruit and vegetables. Findings by Kearns and Mason [66], however, also highlight that whilst ill-health can be associated with a poor intake of fruit and vegetables, it is also associated with a lower intake of take-aways.

Another prevalent issue within this domain, identified in both qualitative and quantitative studies, referred to accessibility, this relates to both shop accessibility and also to the poor accessibility of healthy foods and high accessibility of unhealthy food options such as fast-food [54, 79]. Mobile food stores increased availability of fruit and vegetables in disadvantaged communities and led to an increase intake in fruit and vegetables [64]. Good local services and amenities were also found to be a facilitator [66]. Similarly, altering product placement within supermarkets by allowing more prominent placement of fruits and vegetable and removal of unhealthy foods from checkouts was shown to increase the purchase of healthy products in socioeconomically disadvantaged neighborhoods [91]. However, one study also found that a community garden meant to increase access to fresh fruit and vegetables in a disadvantaged community had very little impact on dietary intake [84]. Similarly, two other studies found that increasing availability of healthier options such as healthier pre-packaged snacks and lower calorie options did not affect food choice for participants from either lower or higher socio-economic status [69, 75].

.

An additional set of variables within this domain referred to living circumstances. Lack of facilities to store, cook or warm food was identified as a barrier [74]. Similarly, eating dinner on one’s lap or on the go was identified as a barrier whereas eating at the dinner table was identified as a facilitator [62]. Household composition could be a barrier or a facilitator, with households with children or single-parent families acting as a barrier, whereas two-parent households acting as a facilitator [66].

This domain also included more general factors such as lack of time which was found to be a barrier by two separate studies [48, 86]. Lack of flavour due to out of season production of fruit and vegetables was also identified as a barrier as was the poor nutritional value of donated food [46, 93]. The chance to attend cultural evets and participate in creative activities were also identified as facilitators [80].

The presence of antisocial behavior in the neighborhood and good internal home quality were identified as both barriers and facilitators [66]. Namely, antisocial behavior in the neighborhood was found to increase the likelihood or fast-food consumption but be associated with increased fruit and vegetable consumption. Similarly, good internal home quality was associated both with higher odds of consuming cakes and snacks but also higher odds of consuming fruits and vegetables [66].

Variables related to upbringing were also mentioned. Upbringing in general and adverse childhood experiences in particular were mentioned as barriers [47, 54], and conversely the presence of always available adults whilst growing up was a facilitator [47]. However, Russell et al. [81] show that this is not a problem specific to disadvantaged communities. They found that childhood violence is a barrier to healthy eating for all socio-economic groups, in the same way that childhood happiness is a facilitator to all.

### Social influences

Similar factors were identified as both a barrier and facilitators. Bonding with others and being part of a community were found to negatively influence healthy eating [48]. This is because unhealthy food options such as take-away consumption can support social relationships. However, support from family was found to positively influence healthy eating [74]. Similarly, the positive role of healthcare professional advice and proxy and collective agency we also found to be facilitators [50]. However, Goodall et al. [69] found that for a group of disadvantaged individuals with cardio-vascular disease, support from lay health trainers did not have significant impact when compared to offering health information leaflets.

### Emotion

No facilitators were identified for this domain. Barriers mostly focused on low mental health and individual’s need for an emotional boost that unhealthy foods can offer e.g. [54]. Fatigue and stress were also mentioned [86]. However, it is worth mentioning that low adult wellbeing was found to be a barrier to healthy eating across all socio-economic positions and thus not specific to disadvantaged individuals [781].

### Behavioral regulation

In this domain, lack of efficacy to engage in a healthy lifestyle was found to be a barrier [50]. Additionally, the need to ration food was found to lead individuals to engage in strategies that lead to unhealthy food related habits such as skipping meals [79]. In terms of facilitators, restricted and budgeted shopping (i.e. having clear shopping objectives and planning purchases, and limited choices decisively either in terms of money, health considerations or both) lead to the purchase of more fruit and vegetables [87]. Additionally, damage control, namely strategies individuals engaged in when wanting unhealthy foods such as managing/ reducing portion size, was found to be a facilitator [48].

In addition to the individual factors described above, four studies also focused on multi-component interventions aimed at targeting several factors places across different TDF domains. The Well-London intervention [77] focused on using traditional health behaviour change activities, improving the local environment, providing cultural activities, and improving employment and training opportunities. This intervention was found to significantly increase the intake of fruit and vegetables. The NHS Health Trainer Service [57] targeted goal setting, action and coping planning but also aimed to make participants aware of environmental triggers to unwanted actions and increase self-efficacy for initiating and maintaining change. The programme also showed a 70% increase in fruit and vegetable intake in disadvantaged individuals. Additionally, the JIGSO young families’ project, offered help to disadvantaged young people from 17 weeks of pregnancy. The project involved women’s antenatal groups, peer-support mother and baby groups, parenting classes and a 6-week healthy relationships course [65]. Findings suggest that the majority of mothers attending the trial improved their diet whilst pregnant as a result of attending the programme. Finally, the intervention from Stead and colleagues [85] focused on marketing price promotion, but also included healthy eating advice and recipe suggestions on the purchase of selected healthier foods by low-income consumers. The intervention led to an increase in purchases for healthy meal deals, and lower fat percentage milk.

## Discussion

The purpose of this study was to identify the barriers and facilitators to healthy eating experienced by disadvantaged individuals in the UK. To the best of our knowledge, this is the first review on barriers and facilitators related to healthy eating specifically for disadvantaged UK adults using a TDF analysis. Findings show that although barriers and facilitators to healthy eating in UK disadvantaged individuals can be identified for most TDF domains, the vast majority can be classed as part of the environmental context and resources domain. Linking our findings to the Behavioural Change Wheel [94], highlights that the environmental context and resources domain relates to the Opportunity part of the COM-B model (‘capability’, ‘opportunity’, ‘motivation’ and ‘behaviour’). This implies that interventions need to focus on modifying factors in the physical environment rather than an individual’s capacity (i.e. capability) or their willingness to change (i.e. motivation). These results align with findings by Hunt and colleagues [41], which show the importance of structural issues and the need for structural change in relation to the diet disadvantaged communities in the UK.

Currently however, most dietary interventions focus on individual decision-making, largely ignoring the effects of environmental cues on human behaviour [95]. Our review challenges this assumption and suggests that environmental factors should also be considered. Despite what our review suggests, the most widely used theoretical framework for behavioural interventions targeting healthy eating is social cognitive theory, which targets self-efficacy and outcome expectancy, both of which are individual factors [96–98]. Therefore, there is an overreliance on the assumption that improving an individual’s nutrition knowledge and skills can lead to them subsequently adopting a healthier diet. This is apparent within the UK, where one of the largest national social marketing campaigns, Change4Life [99], uses information to help promote a healthier lifestyle and where the Levelling-up Agenda [8] highlights its commitments to promoting cooking skills and nutrition education. Whilst our review did identify individual factors such as sense of control and self-efficacy as being influential to healthy eating e.g. [44, 50, 51], it also reveals a nuanced understanding of the factors linked to healthy eating. Cooking skills for example were shown to make a difference for some individuals [48, 74], however findings by Hutchinson et al. [63] imply that such interventions are unlikely to address inequalities. Given the results of our review, policy makes and practitioners need to consider that the offer of cooking classes might not be effective unless participants are also enabled to reliably access healthy ingredients. Interventions that focus on acquiring knowledge should also be considered carefully as other research confirms that disadvantaged individuals are both aware of the need to make changes to their eating habits and willing to do so but struggle nonetheless [78].

Ignoring the importance of environmental factors can lead to poorly developed interventions that can either be ineffective or backfire leaving customers disempowered and eroding policy and public support thus creating more challenges for successful implementation in the future [100–102]. The current review can pinpoint the type of intervention that are more likely to be successful due to the use of the TDF framework and its link to the Behavioural Change Wheel that provides a guide for the selection of intervention functions, policy categories and behaviour change techniques. Specifically, the Behavioural Change Wheel suggests that factors linked to physical opportunity, as identified in our review, should be addressed by interventions targeting restriction, enablement, and environmental restructuring [28].

Interventions based on restriction constrain behaviour by setting rules. In our review, Pechey and colleagues [75] found that removing unhealthy options from disadvantaged communities could have greater impact that increasing access to healthy options. Indeed, interventions that focus on changing the environment in which choices are made through choice-architecture and nudging (i.e. interventions that lead people to do things without it being obvious to them that their behaviour is being shaped) [103] are effective at encouraging healthy eating [104]. For example, convenience enhancements, that is increasing the ease with which consumers can alter their choices has been shown to be one of the most effective nudge strategies [105]. Policy makers in the UK have attempted to implement such an approach through legislation banning multibuy deals on foods and drinks high in fat, salt, or sugar [106]. However, despite evidence about the effectiveness of such an approach, the Government has delayed this legislation, whilst reasoning that individual should have the right to choose what they want to consume in times of economic upheaval [107]. Whilst disappointing, this could be because implementing interventions based on choice architecture can be challenging for policymakers due to negative public opinion [104, 108]. For example, reduction in portion sizes, another behavioural nudge, was found to be one of the most disliked approaches to reducing sugar intake [105]. It could therefore be challenging to use choice architecture to target barriers found in our review such as reliance on processed foods [54] or lack of efficacy to engage in a healthy lifestyle [50]. Encouragingly however, sharing the positives of adopting such an approach can increase public acceptability of interventions [109]. This is why it is also imperative that the intention-behaviour gap (failure to translate intentions into actions) [110] related to healthy eating in disadvantaged individuals is clearly communicated so that there is a shift in the public’s perception that interventions need to focus on individual capacity or motivation. The current review provides further evidence for the intention-behaviour gap related to healthy eating in disadvantaged individuals, as it clearly shows that most barriers and facilitators are outside of individual’s control.

Alternatively, interventions that focus on enablement aim to provide support to improve ability to change [28]. In our review by far the biggest barrier to healthy eating was related to financial issues such as unemployment, inability to work, financial hardship and cost of food [45, 93]. Unfortunately, there has been little effort to address issues related to insecure work which is known to disproportionately affect those from disadvantaged communities [111]. However, it is somewhat encouraging that the UK Government has been considering some recommendations related to interventions that focus on enablement. Two of the recommendations from the National Food Strategy [12] aimed at reducing diet-related inequality focus on affordability and accessibility that can be regarded to be under the enablement umbrella. The recommendation affordability around centres on the expansion of the Healthy Start scheme, and although our review found several studies to support this scheme as a facilitator [60, 67, 70, 72], others have also found this not to make a difference to healthy eating [82]. Additionally, a more recent study using a larger dataset and an appropriate control group also showed that Healthy Start vouchers are unlikely to make an impact on the dietary behaviours of its target population [112]. The recommendation in the National Food Strategy [12] is to expand the financial eligibility and extend the age limit, however this might only be helpful if uptake of the scheme is also improved [70], and if voucher value takes into account rising inflation rates [109]. The second recommendation relates to the implementation of a community programme involving prescriptions of fruit and vegetables, food education and social support. The focus on multiple elements is laudable, as our review also highlights that such interventions can be effective e.g. [57, 77]. Furthermore, prescribing fruits and vegetables could help address financial barriers to healthy eating and thus enable individuals to make healthier choices, this is pertinent given that our review identified high cost of healthy food to be a major barrier e.g. [54, 68, 74, 86, 89]. Currently, however the prescription intervention is being piloted in only two London boroughs and is yet to be rolled out across the UK [113]. Furthermore, these interventions and means tested and only available to families which limits uptake. Other projects that are trying to address these limitations, such as the Fresh Street Community initiative part of the larger FoodSEqual project could provide additional insight into the value of easy and cheap access to fruit and vegetables [114].

The focus on food education however may need further consideration. Our review did highlight that education can act as a barrier and a facilitator to healthy eating, however most studies referred to general education [44, 51, 71, 92]. Only two studies found that food related education could be beneficial to healthy eating [54, 74], however these were studies with a small sample size assessing perceived knowledge rather than actual knowledge. Therefore, on balance, our review highlights that rather than focusing on food knowledge, policy makers might also want to consider raising general educational attainment. Overall, whilst the government has been considering interventions focusing on enabling healthier choices for disadvantaged individuals, more works needs to be done so that individuals can benefit from such interventions.

Finally, interventions aimed at environmental restructuring look at constraining or promoting behaviour by shaping the physical environment [28]. Our review clearly highlights barriers to healthy eating that relate to the physical environment such as takeaway availability [48–50]. One way the Government has tried to address this is via restrictions relating to the accessibility of fast-food outlets in deprived neighbourhoods. However, latest figures suggest that despite proposals to regulate their proliferation through urban planning, only 50% of local authorities have a policy specifically targeting takeaway food outlets, and only 34% focused on health [115]. Worryingly, the effectiveness of urban planning strategies such as restricting new fast-food outlets [116], is being threatened by access to online food delivery services, which appears to be greatest in most deprived areas [117]. This highlights the potential need to develop interventions targeting the use of online delivery services for this target population, Jesse et al. [118], for example show that setting default options and adding social information, are nudges that can help individuals select healthier food options in digital contexts.

Overall, this review highlights important areas where attention could be focused when considering the most effective interventions to improve healthy eating in disadvantaged communities. The systematic approach, use of a TDF to systemize the results and the inclusion of both qualitative and quantitative research are strengths of the paper. Barriers or facilitators could not be identified for several domains, although factors pertaining to these domains have been identified as being of importance in other populations, for example optimism has been shown to positively correlate with healthy eating [119] and higher endorsement of the negative outcomes of obesity is significantly associated with diet quality [120]. This is likely to be due to the retrospective use of the framework on available research results, and highlights gaps in the literature that can be addressed by future research.

Several limitations also need to be acknowledged, given the nature of this review, the quality of the research included was not assessed. Furthermore, when using the TDF reviewers identified a single most relevant domain for each factor, even though domains are not mutually exclusive. The review also did not include grey literature; thus all relevant evidence might have not been identified. Additionally, no analysis of specific sub-populations within the adult population was carried out, for example looking at gender or cultural barriers. We also included several indices to identify disadvantaged individuals but make no comparisons between findings for the different groups in respect to this, future reviews could seek address this in the future.

In conclusion, even though most interventions related to healthy eating focus on individual responsibility, the current review suggests that to encourage healthy eating in disadvantaged individuals the focus should instead be on enabling, restricting, and restructuring the environment. This is because most of the barriers and facilitators that disadvantaged individuals face are linked to the environmental context and the circumstances they live in. We acknowledge that interventions based on choice-architecture, such as the ones suggested in this paper, are difficult to implement, often due to public perception. Thus, we also advise that it is crucial that he public becomes more aware that disadvantaged individuals do desire to eat healthily, but often cannot do so due to the characteristics of the environment they find themselves in. This awareness should make it clear why interventions need to shift away from focusing on motivation and capacity and instead focus on infrastructure and societal frameworks.

### Electronic supplementary material

Below is the link to the electronic supplementary material.

Supplementary Material 1

Supplementary Material 2

Supplementary Material 3

## Data Availability

The dataset used during the current study is available from the corresponding author on reasonable request.

## Footnotes

1 However, although the search included over 60 reviews, none were found to focus on the question of interest, emphasizing the gap in the literature.
