# Supplementary material for: Barriers and facilitators to healthy eating in disadvantaged adults living in the UK: a scoping review
Source: BMC Public Health. 2024 Jul 3;24:1770. doi: 10.1186/s12889-024-19259-2 (PMC11221142; doi:10.1186/s12889-024-19259-2)
Supplement: Supplementary file 2 — Supplementary Material 2 [file 12889_2024_19259_MOESM2_ESM.docx]

Appendix A

Search strategy for all databases

**Search strategy for medline, embase and cinahl:**

(NB when searching CINAHL remove .mp form after food industry and food preferences).

| Population | Search | Terms |
| --- | --- | --- |
|  | S1 | people OR persons OR men OR women OR adult |
|  | S2 | older adults OR older OR elderly OR aged OR aging OR middle aged |
|  | S3 | adult children OR adolescent OR young adult |
|  | S4 | child OR minors OR infant |
|  | S5 | family OR friends OR single person OR parent OR grandparent |
|  | S6 | S1 OR S2 OR S3 OR S4 OR S5 |
|  |  |  |
| Context | S7 | low income OR low-income OR poverty OR poorest OR poverty areas OR social class OR employment insecurity OR precarious employment |
|  | S8 | Factor socioeconomic OR inequalities social OR deprivation OR marginalized OR disadvantaged OR underprivileged OR vulnerable OR vulnerable populations |
|  | S9 | temporary housing OR housing insecurity OR educational disadvantage OR educational level OR cultural diversity |
|  | S10 | S7 OR S8 OR S9 |
|  |  |  |
| Concept | S11 | diet OR diet, healthy OR diet, western OR diet, vegetarian OR infant food OR food OR drink OR beverage OR sustenance OR meal OR snacks |
|  | S12 | nutritious OR food quality OR fresh OR convenience OR product OR packet OR cook OR cooking OR food organic OR food genetically modified |
|  | S13 | home cook OR family meal OR restaurant OR take away OR eat out |
|  | S14 | Diet, food and nutrition |
|  | S15 | nutrients OR nutritive value OR nutritional status OR micronutrients OR vitamins OR dietary fiber OR carbohydrates OR sugars OR proteins OR energy intake OR food ingredients |
|  | S16 | eat OR dine OR consume |
|  | S17 | hunger OR over nutrition OR obesity |
|  | S18 | food assistance OR food deprivation OR food deserts OR food insecurity OR food security OR food supply |
|  | S19 | Food industry.mp |
|  | S20 | Food preferences.mp |
|  | S21 | Health knowledge, attitudes, practice OR attitude to health OR food ways OR food acquisition OR food literacy OR food value |
|  | S22 | S11 OR S12 OR S13 OR S14 OR S15 OR S16 OR S17 OR S18 OR S19 OR S20 OR S21 |
|  | S23 | UK OR United Kingdom OR Britain OR England OR Ireland OR Northern Ireland OR Scotland OR Wales |
|  | S24 | S6 AND S10 AND S22 AND S23 |
|  | S25 | Apply limits - date: 2010 till present, English language, humans. |

**For psycinfo this is the search strategy:**

Search one: people OR persons OR men OR women OR adult OR older adults OR older OR elderly OR aged OR aging OR middle aged OR adult children OR adolescent OR young adult OR child OR minors OR infant OR family OR friends OR single person OR parent OR grandparent

Search two: low income OR low-income OR poverty OR poorest OR poverty areas OR social class OR employment insecurity OR precarious employment OR Factor socioeconomic OR inequalities social OR deprivation OR marginalized OR disadvantaged OR underprivileged OR vulnerable OR vulnerable populations OR temporary housing OR housing insecurity OR educational disadvantage OR educational level OR cultural diversity

Search three: diet OR diet, healthy OR diet, western OR diet, vegetarian OR infant food OR food OR drink OR beverage OR sustenance OR meal OR snacks OR nutritious OR food quality OR fresh OR convenience OR product OR packet OR cook OR cooking OR food organic OR food genetically modified OR home cook OR family meal OR restaurant OR take away OR eat out OR Diet, food and nutrition OR nutrients OR nutritive value OR nutritional status OR micronutrients OR vitamins OR dietary fiber OR carbohydrates OR sugars OR proteins OR energy intake OR food ingredients OR eat OR dine OR consume OR hunger OR over nutrition OR obesity OR food assistance OR food deprivation OR food deserts OR food insecurity OR food security OR food supply OR food industry OR food preferences OR Health knowledge, attitudes, practice OR attitude to health OR food ways OR food acquisition OR food literacy OR food value

Search four: UK OR United Kingdom OR Britain OR England OR Ireland OR Northern Ireland OR Scotland OR Wales

Search five: S1 AND S2 AND S3 AND S4 AND S5

Limit 2010 till present. English language. UK.

**For web of science this is the search strategy:**

#1: ts=(people or adults or men or women or elderly or child) OR ts=(family or parents)

#2: ts=(poverty or deprivation or low income) OR ts=(employment insecurity or housing insecurity or educational disadvantage)

#3:ts=(diets or food or drink or cooking or convenience food or food quality) OR ts=(family meal or eat out or hunger or nutrients or obesity) OR ts=(food insecurity or food ways or food acquisition)

#4: ts=(UK or United Kingdom or Britain or England or Ireland or Northern Ireland or Scotland or Wales)

#5: #1 AND #2 AND #3 AND #4

Limit to UK, English, 2010 to present.
