# Supplementary material for: Barriers and facilitators to healthy eating in disadvantaged adults living in the UK: a scoping review
Source: BMC Public Health. 2024 Jul 3;24:1770. doi: 10.1186/s12889-024-19259-2 (PMC11221142; doi:10.1186/s12889-024-19259-2)
Supplement: Supplementary file 3 — Supplementary Material 3 [file 12889_2024_19259_MOESM3_ESM.docx]

**Appendix C**

**Table 1C**. Excluded records and main exclusion reason

| **Reference** | **Main exclusion reason** |
| --- | --- |
| Adams J, Goffe L, Adamson AJ, Halligan J, O’Brien N, Purves R, Stead M, Stocken D, White M. Prevalence and socio-demographic correlates of cooking skills in UK adults: cross-sectional analysis of data from the UK National Diet and Nutrition Survey. *International Journal of Behavioral Nutrition and Physical Activity*. 2015;12:1-3. | No focus on healthy dietary intake |
| Adams J, Goffe L, Brown T, Lake AA, Summerbell C, White M, Wrieden W, Adamson AJ. Frequency and socio-demographic correlates of eating meals out and take-away meals at home: cross-sectional analysis of the UK national diet and nutrition survey, waves 1–4 (2008–12). *International Journal of Behavioral Nutrition and Physical Activity*. 2015;12:1-9. | Barriers or facilitators not assessed |
| Adams J, White M. Characterisation of UK diets according to degree of food processing and associations with socio-demographics and obesity: cross-sectional analysis of UK National Diet and Nutrition Survey (2008–12). International *Journal of Behavioral Nutrition and Physical Activity*. 2015;12:1-1 | Barriers or facilitators not assessed |
| Adams J, White M. Characterisation of UK diets according to degree of food processing and associations with socio-demographics and obesity: cross-sectional analysis of UK National Diet and Nutrition Survey (2008–12). *International Journal of Behavioral Nutrition and Physical Activity.* 2015;12:1-1. | Barriers or facilitators not assessed |
| Adams J, White M. Prevalence and socio-demographic correlates of time spent cooking by adults in the 2005 UK Time Use Survey. Cross-sectional analysis. *Appetite.* 2015;92:185-91. | No focus on healthy dietary intake |
| Aducovschi D, Dumitrescu R, Gozu B, Gulap M, Şerban C, Otoiu A. Assessing The Influence Of Knowledge, Socio-Economic Status And Self-Consciousness On Nutrition Management. *European Proceedings of Social and Behavioural Sciences.* 2018;42:130-139. | No focus on healthy dietary intake |
| Aljawad A, Morgan MZ, Rees JS, Fairchild R. The availability of novelty sweets within high school localities. *British Dental Journal.* 2016;220(11):575-9. | No focus on healthy dietary intake |
| Allan JL, Johnston M, Campbell N. Snack purchasing is healthier when the cognitive demands of choice are reduced: A randomized controlled trial. *Health Psychology.* 2015;34(7):750. | No results for disadvantaged groups |
| Almoosawi S, Winter J, Prynne CJ, Hardy R, Stephen AM. Daily profiles of energy and nutrient intakes: are eating profiles changing over time?. *European Journal of Clinical Nutrition.* 2012;66(6):678-86. | Barriers or facilitators not assessed |
| Alwan NA, Greenwood DC, Simpson NA, McArdle HJ, Godfrey KM, Cade JE. Dietary iron intake during early pregnancy and birth outcomes in a cohort of British women. *Human Reproduction*. 2011;26(4):911-9. | Barriers or facilitators not assessed |
| An R. Effectiveness of subsidies in promoting healthy food purchases and consumption: a review of field experiments. *Public Health Nutrition*. 2013;16(7):1215-28. | No UK specific results |
| Andrews EJ, Symon A, Anderson AS. ‘I didn't know why you had to wait’: an evaluation of NHS infant‐feeding workshops amongst women living in areas of high deprivation. *Journal of Human Nutrition and Dietetics*. 2015;28(6):558-67. | No focus on healthy dietary intake |
| Appleton KM, McGill R, Neville C, Woodside JV. Barriers to increasing fruit and vegetable intakes in the older population of Northern Ireland: low levels of liking and low awareness of current recommendations. *Public Health Nutrition*. 2010;13(4):514-21. | Does not link variables of interest |
| Appleton KM, McKeown PP, Woodside JV. Energy compensation in the real world: Good compensation for small portions of chocolate and biscuits over short time periods in complicit consumers using commercially available foods. *Appetite*. 2015;85:104-10. | No results for disadvantaged groups |
| Astbury CC, Penney TL, Adams J. Home-prepared food, dietary quality and socio-demographic factors: a cross-sectional analysis of the UK National Diet and nutrition survey 2008–16. *The International Journal of Behavioral Nutrition and Physical Activity.* 2019;16. | Barriers or facilitators not assessed |
| Aston LM, Smith JN, Powles JW. Meat intake in Britain in relation to other dietary components and to demographic and risk factor variables: analyses based on the National Diet and Nutrition Survey of 2000/2001*. Journal of Human Nutrition and Dietetics*. 2013;26(1):96-106. | Does not link variables of interest |
| Atkins JL, Ramsay SE, Whincup PH, Morris RW, Lennon LT, Wannamethee SG. Diet quality in older age: the influence of childhood and adult socio-economic circumstances. British Journal of Nutrition. 2015;113(9):1441-52. | Does not link variables of interest |
| Atkins JL, Whincup PH, Morris RW, Wannamethee SG. Dietary patterns in older British men: the influence of early life social circumstances and area of residence. *Proceedings of the Nutrition Society*. 2013; 72(OCE4):E251. | Abstract only |
| Ball K, Lamb KE, Costa C, Cutumisu N, Ellaway A, Kamphuis CB, Mentz G, Pearce J, Santana P, Santos R, Schulz AJ. Neighbourhood socioeconomic disadvantage and fruit and vegetable consumption: a seven countries comparison. *International Journal of Behavioral Nutrition and Physical Activity*. 2015;12:1-3. | Barriers or facilitators not assessed |
| Barfoot KL, Forster R, Lamport DJ. Mental health in new mothers: a randomised controlled study into the effects of dietary flavonoids on mood and perceived quality of life. *Nutrients*. 2021;13(7):2383. | No results for disadvantaged groups |
| Barrett P, Imamura F, Brage S, Griffin SJ, Wareham NJ, Forouhi NG. Sociodemographic, lifestyle and behavioural factors associated with consumption of sweetened beverages among adults in Cambridgeshire, UK: the Fenland Study. *Public Health Nutrition*. 2017;20(15):2766-77. | Does not link variables of interest |
| Barton KL, Chambers S, Anderson AS, Wrieden WL. Time to address the double inequality of differences in dietary intake between Scotland and England. *British Journal of Nutrition*. 2018;120(2):220-6. | Barriers or facilitators not assessed |
| Barton KL, Wrieden WL, Sherriff A, Armstrong J, Anderson AS. Trends in socio-economic inequalities in the Scottish diet: 2001–2009. *Public Health Nutrition*. 2015;18(16):2970-80. | Barriers or facilitators not assessed |
| Bates RL, Craig LC, Watts D. UK residents with low incomes and healthy diets: in search of exemplars. *Proceedings of the Nutrition Society*. 2020;79(OCE3):E742. | No results for disadvantaged groups |
| Baybutt M, Dooris M, Farrier A. Growing health in UK prison settings. *Health Promotion International*. 2019;34(4):792-802. | No results for disadvantaged groups |
| Beacom E, Furey S, Hollywood L, Humphreys P. Investigating food insecurity measurement globally to inform practice locally: a rapid evidence review. *Critical Reviews in Food Science and Nutrition*. 2021;61(20):3319-39. | No focus on healthy dietary intake |
| Bellis MA, Lowey H, Leckenby N, Hughes K, Harrison D. Adverse childhood experiences: retrospective study to determine their impact on adult health behaviours and health outcomes in a UK population. *Journal of Public Health*. 2014;36(1):81-91. | No results for disadvantaged groups |
| Best M, Papies EK. Lower socioeconomic status is associated with higher intended consumption from oversized portions of unhealthy food. *Appetite.* 2019;140:255-68. | No focus on healthy dietary intake; focused on intended consumption not actual consumption |
| Beydoun MA, Fanelli-Kuczmarski MT, Poti J, Allen A, Beydoun HA, Evans MK, Zonderman AB. Longitudinal change in the diet's monetary value is associated with its change in quality and micronutrient adequacy among urban adults. *PloS One*. 2018;13(10):e0204141.. | No UK specific results |
| Birch J, Petty R, Hooper L, Bauld L, Rosenberg G, Vohra J. Clustering of behavioural risk factors for health in UK adults in 2016: a cross-sectional survey. *Journal of Public Health*. 2019;41(3):e226-36. | Barriers or facilitators not assessed |
| Blitstein JL, Guthrie JF, Rains C. Low-income parents’ use of front-of-package nutrition labels in a virtual supermarket. *Journal of Nutrition Education and Behavior*. 2020;52(9):850-8. | No UK specific results |
| Bodicoat DH, Carter P, Comber A, Edwardson C, Gray LJ, Hill S, Webb D, Yates T, Davies MJ, Khunti K. Is the number of fast-food outlets in the neighbourhood related to screen-detected type 2 diabetes mellitus and associated risk factors?. *Public Health Nutrition*. 2015;18(9):1698-705. | No focus on healthy dietary intake |
| Bokhari F, Derbyshire EJ, Li W, Brennan CS. Can an iron-rich staple food help women to achieve dietary targets in pregnancy?. *International Journal of Food Sciences and Nutrition*. 2012;63(2):199-207. | No results for disadvantaged groups |
| Bratanova B, Loughnan S, Klein O, Claassen A, Wood R. Poverty, inequality, and increased consumption of high calorie food: Experimental evidence for a causal link. *Appetite.* 2016;100:162-71. | No results for disadvantaged groups |
| Brough L, Rees GA, Crawford MA, Morton RH, Dorman EK. Effect of multiple-micronutrient supplementation on maternal nutrient status, infant birth weight and gestational age at birth in a low-income, multi-ethnic population. *British Journal of Nutrition*. 2010;104(3):437-45. | Barriers or facilitators not assessed |
| Browne C, Twefik I. Hidden Hungers of Camden: A Pilot Intervention unraveling the double burden of Malnutrition Among Ethnic Minority Women living in Deprivation. *Proceedings of the Nutrition Society.* 2020;79(OCE1):E25. | Abstract only |
| Buckland NJ, Finlayson G, Edge R, Hetherington MM. Resistance reminders: Dieters reduce energy intake after exposure to diet-congruent food images compared to control non-food images*. Appetite*. 2014;73:189-96. | No results for disadvantaged groups |
| Bull ER, Dombrowski SU, McCleary N, Johnston M. Are interventions for low-income groups effective in changing healthy eating, physical activity and smoking behaviours? A systematic review and meta-analysis. *BMJ Open.* 2014;4(11):e006046. | No UK specific results |
| Burgoine T, Alvanides S, Lake AA. Assessing the obesogenic environment of North East England. *Health & Place.* 2011;17(3):738-47. | Does not link variables of interest |
| Burgoine T, Forouhi NG, Griffin SJ, Brage S, Wareham NJ, Monsivais P. Does neighborhood fast-food outlet exposure amplify inequalities in diet and obesity? A cross-sectional study. *The American Journal of Clinical Nutrition.* 2016;103(6):1540-7. | No adequate SES measure |
| Burgoine T, Sarkar C, Webster C, Monsivais P. Interplay of takeaway food outlet exposure and income on diet and obesity: a cross-sectional study in UK Biobank. *The Lancet*. 2016;388:S28. | Abstract only |
| Cairns P, Ozakinci G, Perrett DI. Reactions to an online demonstration of the effect of Increased fruit and vegetable consumption on appearance: survey study. *Journal of Medical Internet Research*. 2020;22(7):e15726. | No focus on healthy dietary intake |
| Caraher M, Lloyd S, Lawton J, Singh G, Horsley K, Mussa F. A tale of two cities: A study of access to food, lessons for public health practice. Health Education Journal. 2010;69(2):200-10. | No focus on healthy dietary intake |
| Cetateanu A, Jones A. Understanding the relationship between food environments, deprivation and childhood overweight and obesity: evidence from a cross sectional England-wide study. *Health & Place*. 2014;27:68-76. | No focus on healthy dietary intake |
| Chattopadhyay K, Akagwire U, Biswas M, Moore R, Rajania G, Lewis S. Role of lifestyle behaviours in the ethnic pattern of poor health outcomes in Leicester, England: analysis of a survey data set. *Public Health*. 2019;170:122-8. | No results for disadvantaged groups |
| Chattopadhyay K, Biswas M, Moore R. NHS health check and healthy lifestyle in Leicester, England: analysis of a survey dataset. *Perspectives in Public Health*. 2020;140(1):27-37. | Does not link variables of interest |
| Chawner LR, Blundell-Birtill P, Hetherington MM. Predictors of vegetable consumption in children and adolescents: analyses of the UK National Diet and Nutrition Survey (2008–2017). *British Journal of Nutrition*. 2021;126(2):295-306. | Majority of participants under 18 |
| Conklin AI, Forouhi NG, Brunner EJ, Monsivais P. Persistent financial hardship, 11‐year weight gain, and health behaviors in the W hitehall II study. *Obesity*. 2014;22(12):2606-12. | No focus on healthy dietary intake |
| Conklin AI, Forouhi NG, Suhrcke M, Surtees P, Wareham NJ, Monsivais P. Variety more than quantity of fruit and vegetable intake varies by socioeconomic status and financial hardship. Findings from older adults in the EPIC cohort. *Appetite.* 2014;83:248-55. | Barriers or facilitators not assessed |
| Craig LC, McNeill G, Masson LF, Macdiarmid JI. Diet Quality Index in children in Scotland: associations with age, sex, socio-economic deprivation and obesity. *Proceedings of the Nutrition Society.* 2016 Jan;75(OCE3):E185. | Abstract only |
| Craig LC, McNeill G, Masson LF, Macdiarmid JI. Diet Quality Index in children in Scotland: associations with age, sex, socio-economic deprivation and obesity*. Proceedings of the Nutrition Society.* 2016;75(OCE3):E185. | Abstract only |
| Craigie AM, Macleod M, Barton KL, Treweek S, Anderson AS. Supporting postpartum weight loss in women living in deprived communities: design implications for a randomised control trial. *European Journal of Clinical Nutrition.* 2011;65(8):952-8. | No focus on healthy dietary intake |
| Cross-Bardell L, George T, Bhoday M, Tuomainen H, Qureshi N, Kai J. Perspectives on enhancing physical activity and diet for health promotion among at-risk urban UK South Asian communities: a qualitative study. *BMJ Open*. 2015;5(2):e007317. | No focus on healthy dietary intake |
| Darling AL, Blackbourn DJ, Ahmadi KR, Lanham-New SA. Vitamin D supplement use and associated demographic, dietary and lifestyle factors in 8024 South Asians aged 40–69 years: analysis of the UK Biobank cohort. *Public Health Nutrition*. 2018;21(14):2678-88. | Does not link variables of interest |
| Darzi J, Frost GS, Montaser R, Yap J, Robertson MD. Influence of the tolerability of vinegar as an oral source of short-chain fatty acids on appetite control and food intake. *International Journal of Obesity*. 2014;38(5):675-81. | No focus on healthy dietary intake |
| Davidson EM, Liu JJ, Bhopal RA, White M, Johnson MR, Netto G, Wabnitz C, Sheikh A. Behavior change interventions to improve the health of racial and ethnic minority populations: a tool kit of adaptation approaches. *The Milbank Quarterly.* 2013;91(4):811-51. | No focus on healthy dietary intake |
| de Brito‐Ashurst I, Perry L, Sanders TA, Thomas JE, Yaqoob MM, Dobbie H. Barriers and facilitators of dietary sodium restriction amongst Bangladeshi chronic kidney disease patients. *Journal of Human Nutrition and Dietetics.* 2011;24(1):86-95. | No results for disadvantaged groups |
| Deeming C. Food and nutrition security at risk in later life: evidence from the United Kingdom Expenditure & Food Survey*. Journal of Social Policy*. 2011;40(3):471-92. | Barriers or facilitators not assessed |
| Defeyter MA, Graham PL, Prince K. A qualitative evaluation of holiday breakfast clubs in the UK: views of adult attendees, children, and staff. *Frontiers in Public Health*. 2015;3:155158. | No focus on healthy dietary intake |
| Del Corral P, Bryan DR, Garvey WT, Gower BA, Hunter GR. Dietary adherence during weight loss predicts weight regain. *Obesity.* 2011;19(6):1177-81. | No UK specific results |
| Denniss RJ, Barker LA, Day CJ. Improvement in cognition following double-blind randomized micronutrient interventions in the general population. *Frontiers in Behavioral Neuroscience*. 2019;13:115. | No results for disadvantaged groups |
| Dewar DL, Morgan PJ, Plotnikoff RC, Okely AD, Collins CE, Batterham M, Callister R, Lubans DR. The nutrition and enjoyable activity for teen girls study: a cluster randomized controlled trial. *American Journal of Preventive Medicine.* 2013;45(3):313-7. | No UK specific results |
| Dolan A. ‘You can’t ask for a Dubonnet and lemonade!’: working class masculinity and men’s health practices. *Sociology of Health & Illness*. 2011;33(4):586-601. | No focus on healthy dietary intake |
| Douglas, F., Sapko, J., Kiezebrink, K. and Kyle, J., 2015. Resourcefulness, desperation, shame, gratitude and powerlessness: common themes emerging from a study of food bank use in Northeast Scotland. *AIMS Public Health*, 2(3), p.297. | No focus on healthy dietary intake |
| Ducrot P, Julia C, Méjean C, Kesse-Guyot E, Touvier M, Fezeu LK, Hercberg S, Péneau S. Impact of different front-of-pack nutrition labels on consumer purchasing intentions: a randomized controlled trial. *American Journal of Preventive Medicine.* 2016;50(5):627-36. | No UK specific results |
| Ejebu OZ, Whybrow S, Mckenzie L, Dowler E, Garcia AL, Ludbrook A, Barton KL, Wrieden WL, Douglas F. What can secondary data tell us about household food insecurity in a high-income country context?. *International Journal of Environmental Research and Public Health*. 2019 ;16(1):82. | Barriers or facilitators not assessed |
| Ellaway A, Macdonald L, Lamb K, Thornton L, Day P, Pearce J. Do obesity-promoting food environments cluster around socially disadvantaged schools in Glasgow, Scotland?. *Health & Place*. 2012;18(6):1335-40. | No focus on healthy dietary intake |
| Ensaff H, Canavon C, Crawford R, Barker ME. A qualitative study of a food intervention in a primary school: Pupils as agents of change. *Appetite*. 2015;95:455-65. | No results for disadvantaged groups |
| Eshareturi C, Wareham A, Rattray M, Haith-Cooper M, McCarthy R. An exploration of the impact of SARS-CoV-2 (COVID-19) restrictions on marginalised groups in the UK. *Public Health*. 2021;197:6-10. | Barriers or facilitators not assessed |
| Everson-Hock ES, Johnson M, Jones R, Woods HB, Goyder E, Payne N, Chilcott J. Community-based dietary and physical activity interventions in low socioeconomic groups in the UK: a mixed methods systematic review. *Preventive Medicine.* 2013;56(5):265-72. | Barriers or facilitators not assessed |
| Fava FR, Gitau R, Griffin BA, Gibson GR, Tuohy KM, Lovegrove JA. The type and quantity of dietary fat and carbohydrate alter faecal microbiome and short-chain fatty acid excretion in a metabolic syndrome ‘at-risk’population. *International Journal of Obesity*. 2013;37(2):216-23. | No results for disadvantaged groups |
| Flaherty SJ, McCarthy MB, Collins AM, McCafferty C, McAuliffe FM. A phenomenological exploration of change towards healthier food purchasing behaviour in women from a lower socioeconomic background using a health app. *Appetite*. 2020;147:104566. | No UK specific results |
| Forwood SE, Ahern AL, Hollands GJ, Ng YL, Marteau TM. Priming healthy eating. You can't prime all the people all of the time. *Appetite*. 2015;89:93-102. | No results for disadvantaged groups |
| Foster HM, Celis-Morales CA, Nicholl BI, Petermann-Rocha F, Pell JP, Gill JM, O'Donnell CA, Mair FS. The effect of socioeconomic deprivation on the association between an extended measurement of unhealthy lifestyle factors and health outcomes: a prospective analysis of the UK Biobank cohort. *The Lancet Public Health*. 2018;3(12):e576-85. | Does not link variables of interest |
| Fraser LK, Edwards KL, Tominitz M, Clarke GP, Hill AJ. Food outlet availability, deprivation and obesity in a multi-ethnic sample of pregnant women in Bradford, UK. *Social Science & Medicine*. 2012;75(6):1048-56. | No focus on healthy dietary intake |
| Frost CJ, Pelham-Burn SE, Russell JM, Barker ME. Improving the nutritional quality of charitable meals for homeless and vulnerable adults: A mixed method study of two meals services in a large English city. *Journal of Hunger & Environmental Nutrition*. 2016;11(1):14-28. | No focus on healthy dietary intake |
| Garthwaite K. Stigma, shame and ‘people like us’: an ethnographic study of foodbank use in the UK. *Journal of Poverty and Social Justice.* 2016;24(3):277-89. | No focus on healthy dietary intake |
| Gazeley I, Newell A, Reynolds K, Rufrancos H. How hungry were the poor in late 1930s Britain?. *The Economic History Review*. 2022;75(1):80-110. | No results for disadvantaged groups |
| Gell L, Meier P. The nature and strength of the relationship between expenditure on alcohol and food: An analysis of adult‐only households in the UK. *Drug and Alcohol Review*. 2012;31(4):422-30. | Does not link variables of interest |
| Ghawi SK, Rowland I, Methven L. Enhancing consumer liking of low salt tomato soup over repeated exposure by herb and spice seasonings*. Appetite*. 2014;81:20-9. | No focus on healthy dietary intake |
| Gibson LM, Nolan J, Littlejohns TJ, Mathieu E, Garratt S, Doherty N, Petersen S, Harvey NC, Sellors J, Allen NE, Wardlaw JM. Factors associated with potentially serious incidental findings and with serious final diagnoses on multi-modal imaging in the UK Biobank Imaging Study: A prospective cohort study*. PLoS One*. 2019;14(6):e0218267. | No focus on healthy dietary intake |
| Golley RK, Smithers LG, Mittinty MN, Brazionis L, Emmett P, Northstone K, Campbell K, McNaughton SA, Lynch JW. An index measuring adherence to complementary feeding guidelines has convergent validity as a measure of infant diet quality. *The Journal of Nutrition*. 2012;142(5):901-8. | Barriers or facilitators not assessed |
| Gorgulho BM, Pot GK, Sarti FM, Marchioni DM. Main meal quality in Brazil and United Kingdom: Similarities and differences. *Appetite*. 2017;111:151-7. | No results for disadvantaged groups |
| Graham H, Hutchinson J, Law C, Platt L, Wardle H. Multiple health behaviours among mothers and partners in England: clustering, social patterning and intra-couple concordance. *SSM-population health*. 2016;2:824-33. | Does not link variables of interest |
| Green MA, Subramanian SV, Strong M, Cooper CL, Loban A, Bissell P. ‘Fish out of water’: a cross-sectional study on the interaction between social and neighbourhood effects on weight management behaviours. *International Journal of Obesity*. 2015;39(3):535-41. | Barriers or facilitators not assessed |
| Griffin T, Sun Y, Sidhu M, Adab P, Burgess A, Collins C, Daley A, Entwistle A, Frew E, Hardy P, Hurley K. Healthy Dads, Healthy Kids UK, a weight management programme for fathers: feasibility RCT. *BMJ Open*. 2019;9(12):e033534. | No focus on healthy dietary intake |
| Haggarty P, Campbell DM, Knox S, Horgan GW, Hoad G, Boulton E, McNeill G, Wallace AM. Vitamin D in pregnancy at high latitude in Scotland*. British Journal of Nutrition*. 2013;109(5):898-905. | No results for disadvantaged groups |
| Hamer M, Mishra GD. Dietary patterns and cardiovascular risk markers in the UK Low Income Diet and Nutrition Survey. *Nutrition, Metabolism and Cardiovascular Diseases.* 2010;20(7):491-7. | Barriers or facilitators not assessed |
| Hancock C, Clarke SK, Stevens DE. Supporting individuals’ healthy eating requires genuine engagement with communities. *Nutrition Bulletin*. 2019;44(1):92-9. | Not primary research |
| Hannon EM, Learner B. The nutritional impact of breakfast programs in the UK. *Proceedings of the Nutrition Society.* 2016;75(OCE3):E236. | Barriers or facilitators not assessed |
| Harvey K. “When I go to bed hungry and sleep, I'm not hungry”: children and parents' experiences of food insecurity. *Appetite*. 2016;99:235-44. | Barriers or facilitators not assessed |
| Harvey-Golding L, Donkin LM, Blackledge J, Defeyter MA. Universal free school breakfast: a qualitative model for breakfast behaviors. *Frontiers in Public Health*. 2015;3:145551.. | Does not link variables of interest |
| Hawkesworth S, Silverwood RJ, Armstrong B, Pliakas T, Nanchahal K, Sartini C, Amuzu A, Wannamethee G, Atkins J, Ramsay SE, Casas JP. Investigating the importance of the local food environment for fruit and vegetable intake in older men and women in 20 UK towns: a cross-sectional analysis of two national cohorts using novel methods*. International Journal of Behavioral Nutrition and Physical Activity*. 2017;14:1-4. | Does not link variables of interest |
| Henshaw P. The latest findings from the Millenium Cohort Study. *British Journal of School Nursing*. 2014;9(10):498-500. | Majority of participants under 18 |
| Heslehurst N, Flynn AC, Ngongalah L, McParlin C, Dalrymple KV, Best KE, Rankin J, McColl E. Diet, physical activity and gestational weight gain patterns among pregnant women living with obesity in the North East of England: the GLOWING pilot trial. *Nutrients.* 2021;13(6):1981. | Does not link variables of interest |
| Hobbs M, Green M, Roberts K, Griffiths C, McKenna J. Reconsidering the relationship between fast-food outlets, area-level deprivation, diet quality and body mass index: an exploratory structural equation modelling approach. *Journal of Epidemiology and Community Health.* 2019;73(9):861-6. | Barriers or facilitators not assessed |
| Holmes BA, Kaffa N, Campbell K, Sanders TA. The contribution of breakfast cereals to the nutritional intake of the materially deprived UK population. *European Journal of Clinical Nutrition.* 2012;66(1):10-7. | Barriers or facilitators not assessed |
| Horsley JA, Absalom KA, Akiens EM, Dunk RJ, Ferguson AM. The proportion of unhealthy foodstuffs children are exposed to at the checkout of convenience supermarkets. *Public Health Nutrition.* 2014;17(11):2453-8. | No results for disadvantaged groups |
| Huang P, O’Keeffe M, Elia C, Karamanos A, Goff LM, Maynard M, Cruickshank JK, Harding S. Fruit and vegetable consumption and mental health across adolescence: evidence from a diverse urban British cohort study. *International Journal of Behavioral Nutrition and Physical Activity*. 2019;16:1-3. | Does not link variables of interest |
| Hufton E, Raven J. Exploring the infant feeding practices of immigrant women in the North West of England: a case study of asylum seekers and refugees in Liverpool and Manchester. *Maternal & Child Nutrition*. 2016;12(2):299-313. | No focus on healthy dietary intake |
| Hutchinson J, Rippin HL, Jewell J, Breda JJ, Cade JE. Comparison of high and low trans-fatty acid consumers: analyses of UK National Diet and Nutrition Surveys before and after product reformulation. *Public Health Nutrition*. 2018;21(3):465-79. | Barriers or facilitators not assessed |
| Irz X, Fratiglioni L, Kuosmanen N, Mazzocchi M, Modugno L, Nocella G, Shakersain B, Traill WB, Xu W, Zanello G. Sociodemographic determinants of diet quality of the EU elderly: a comparative analysis in four countries. *Public Health Nutrition*. 2014;17(5):1177-89. | Does not link variables of interest |
| Jackson RA, Stotland NE, Caughey AB, Gerbert B. Improving diet and exercise in pregnancy with Video Doctor counseling: a randomized trial. *Patient Education and Counseling*. 2011;83(2):203-9. | No UK specific results |
| James R, James LJ, Clayton DJ. Anticipation of 24 h severe energy restriction increases energy intake and reduces physical activity energy expenditure in the prior 24 h, in healthy males. *Appetite*. 2020;152:104719. | No results for disadvantaged groups |
| Jarosz E. Class and eating: Family meals in Britain. *Appetite*. 2017;116:527-35. | Barriers or facilitators not assessed |
| Jenkins M. An assessment of homeless families' diet and nutrition. *Community Practitioner*. 2014;87(4). | Does not link variables of interest |
| Ji C, Cappuccio FP. Socioeconomic inequality in salt intake in Britain 10 years after a national salt reduction programme. *BMJ Open*. 2014;4(8):e005683. | Barriers or facilitators not assessed |
| Jofre-Bonet M, Serra-Sastre V, Vandoros S. The impact of the Great Recession on health-related risk factors, behaviour and outcomes in England. *Social Science & Medicine*. 2018;197:213-25. | Does not link variables of interest |
| Johnson L, Toumpakari Z, Papadaki A. Social gradients and physical activity trends in an obesogenic dietary pattern: Cross-sectional analysis of the UK National Diet and Nutrition Survey 2008–2014. *Nutrients*. 2018;10(4):388. | Barriers or facilitators not assessed |
| Keeble M, Adams J, White M, Summerbell C, Cummins S, Burgoine T. Correlates of English local government use of the planning system to regulate hot food takeaway outlets: a cross-sectional analysis. *International Journal of Behavioral Nutrition and Physical Activity.* 2019;16:1-2. | No focus on healthy dietary intake |
| Kerr DA, Harray AJ, Pollard CM, Dhaliwal SS, Delp EJ, Howat PA, Pickering MR, Ahmad Z, Meng X, Pratt IS, Wright JL. The connecting health and technology study: a 6-month randomized controlled trial to improve nutrition behaviours using a mobile food record and text messaging support in young adults. *International Journal of Behavioral Nutrition and Physical Activity.* 2016;13:1-4. | No UK specific results |
| Khaled K, Hundley V, Almilaji O, Koeppen M, Tsofliou F. A priori and a posteriori dietary patterns in women of childbearing age in the UK*. Nutrients*. 2020 Sep;12(10):2921. | Barriers or facilitators not assessed |
| Khaled K, Hundley V, and Tsofliou F. Poor dietary quality and patterns are associated with higher perceived stress among women of reproductive age in the UK. *Nutrients*, 2021, 13(8), p.2588. | No results for disadvantaged groups |
| Kininmonth AR, Smith AD, Llewellyn CH, Fildes A. Socioeconomic status and changes in appetite from toddlerhood to early childhood. *Appetite*. 2020;146:104517. | Barriers or facilitators not assessed |
| Knight A, Brannen J, O'Connell R, Hamilton L. How do children and their families experience food poverty according to UK newspaper media 2006–15?. *Journal of Poverty and Social Justice.* 2018;26(2):207-23. | No focus on healthy dietary intake |
| Korani M, Rea DM, King PF, Brown AE. Significant differences in maternal child‐feeding style between ethnic groups in the UK: the role of deprivation and parenting styles. *Journal of Human Nutrition and Dietetics*. 2018;31(5):625-33. | No focus on healthy dietary intake |
| Lakshman R, McConville A, How S, Flowers J, Wareham N, Cosford P. Association between area-level socioeconomic deprivation and a cluster of behavioural risk factors: cross-sectional, population-based study. *Journal of Public Health*. 2011;33(2):234-45. | Barriers or facilitators not assessed |
| Lakshman RR, Sharp SJ, Ong KK, Forouhi NG. A novel school-based intervention to improve nutrition knowledge in children: cluster randomised controlled trial. *BMC Public Health*. 2010;10:1-9. | No results for disadvantaged groups |
| Lam MC, Adams J. Association between home food preparation skills and behaviour, and consumption of ultra-processed foods: Cross-sectional analysis of the UK National Diet and nutrition survey (2008–2009). *International Journal of Behavioral Nutrition and Physical Activity*. 2017;14:1-7. | Does not link variables of interest |
| Laverty AA, Magee L, Monteiro CA, Saxena S, Millett C. Sugar and artificially sweetened beverage consumption and adiposity changes: National longitudinal study. *International Journal of Behavioral Nutrition and Physical Activity*. 2015;12:1-0. | No results for disadvantaged groups |
| Lazzarino AI, Yiengprugsawan V, Seubsman SA, Steptoe A, Sleigh AC. The associations between unhealthy behaviours, mental stress, and low socio-economic status in an international comparison of representative samples from Thailand and England. *Globalization and Health*. 2014;10:1-8. | No focus on healthy dietary intake |
| Leigh JP, Leigh WA, Du J. Minimum wages and public health: a literature review. Preventive medicine. 2019;118:122-34. | No focus on healthy dietary intake |
| Levin KA, Kirby J, Currie C, Inchley J. Trends in adolescent eating behaviour: a multilevel cross-sectional study of 11–15 year olds in Scotland, 2002–2010. *Journal of Public Health*. 2012;34(4):523-31. | Barriers or facilitators not assessed |
| Levin KA, Kirby J. Irregular breakfast consumption in adolescence and the family environment: Underlying causes by family structure. *Appetite*. 2012;59(1):63-70. | Does not link variables of interest |
| Li Y, Dai Q, Ekperi LI, Dehal A, Zhang J. Fish consumption and severely depressed mood, findings from the first national nutrition follow-up study. *Psychiatry Research*. 2011;190(1):103-9. | No results for disadvantaged groups |
| Linton MJ, Jones T, Owen-Smith A, Payne RA, Coast J, Glynn J, Hollingworth W. Breaking bread: examining the impact of policy changes in access to state-funded provisions of gluten-free foods in England. *BMC Medicine*. 2018;16:1-9. | No focus on healthy dietary intake |
| Lloyd S, Lawton J, Caraher M, Singh G, Horsley K, Mussa F. A tale of two localities: Healthy eating on a restricted income. *Health Education Journal*. 2011;70(1):48-56. | No focus on healthy dietary intake |
| Lubans DR, Plotnikoff RC, Morgan PJ, Dewar D, Costigan S, Collins CE. Explaining dietary intake in adolescent girls from disadvantaged secondary schools. A test of Social Cognitive Theory. *Appetite*. 2012;58(2):517-24. | No UK specific results |
| Ma Y, He FJ, Yin Y, Hashem KM, MacGregor GA. Gradual reduction of sugar in soft drinks without substitution as a strategy to reduce overweight, obesity, and type 2 diabetes: a modelling study. *The Lancet Diabetes & Endocrinology*. 2016;4(2):105-14. | No focus on healthy dietary intake |
| Macdonald L, Olsen JR, Shortt NK, Ellaway A. Do ‘environmental bads’ such as alcohol, fast food, tobacco, and gambling outlets cluster and co-locate in more deprived areas in Glasgow City, Scotland?. *Health & Place*. 2018;51:224-31. | No focus on healthy dietary intake |
| Maguire ER, Burgoine T, Penney TL, Forouhi NG, Monsivais P. Does exposure to the food environment differ by socioeconomic position? Comparing area-based and person-centred metrics in the Fenland Study, UK*. International Journal of Health Geographics*. 2017;16:1-4. | No focus on healthy dietary intake |
| Maguire ER, Monsivais P. Socio-economic dietary inequalities in UK adults: an updated picture of key food groups and nutrients from national surveillance data. *British Journal of Nutrition*. 2015;113(1):181-9. | Barriers or facilitators not assessed |
| Mak TN, Prynne CJ, Cole D, Fitt E, Bates B, Stephen AM. Patterns of sociodemographic and food practice characteristics in relation to fruit and vegetable consumption in children: results from the UK National Diet and Nutrition Survey Rolling Programme (2008–2010). *Public Health Nutrition*. 2013v;16(11):1912-23. | Does not link variables of interest |
| Mann KD, Pearce MS, McKevith B, Thielecke F, Seal CJ. Low whole grain intake in the UK: results from the National Diet and Nutrition Survey rolling programme 2008–11. *British Journal of Nutrition*. 2015;113(10):1643-51. | Barriers or facilitators not assessed |
| Marcano-Olivier M, Pearson R, Ruparell A, Horne PJ, Viktor S, Erjavec M. A low-cost Behavioural Nudge and choice architecture intervention targeting school lunches increases children’s consumption of fruit: a cluster randomised trial. *International Journal of Behavioral Nutrition and Physical Activity*. 2019;16:1-9. | No results for disadvantaged groups |
| Marquis L, Oliver MC, Gould E, Hyland M, Kuri V. A focus group exploration of primary school children's perceptions and experiences of fruit and vegetables. *Journal of Human Nutrition and Dietetics*. 2011;24(3):292-. | Abstract only |
| Marteau TM, Jebb SA, Lewis HB, Wei Y, Higgins JP, Ogilvie D. Portion, package or tableware size for changing selection and consumption of food, alcohol and tobacco. *Cochrane database of systematic reviews.* 2015(9). | No UK specific results |
| Masic U, Yeomans MR. Does acute or habitual protein deprivation influence liking for monosodium glutamate?. *Physiology & Behavior*. 2017;171:79-86. | No results for disadvantaged groups |
| Masson LF, Barton KL, Wrieden WL. The Scottish diet is poorer in households purchasing tobacco products: analysis of Living Costs and Food Survey data from 2001–2012. *Proceedings of the Nutrition Society*. 2016;75(OCE3):E145. | Abstract only |
| Masson LF, Blackburn A, Sheehy C, Craig LC, Macdiarmid JI, Holmes BA, McNeill G. Sugar intake and dental decay: results from a national survey of children in Scotland. *British Journal of Nutrition.* 2010;104(10):1555-64. | Does not link variables of interest |
| Mayurasakorn K, Pinsawas B, Mongkolsucharitkul P, Sranacharoenpong K, Damapong SN. School closure, COVID‐19 and lunch programme: Unprecedented undernutrition crisis in low‐middle income countries*. Journal of Paediatrics and Child Health*. 2020;56(7):1013-7. | No UK specific results |
| McCabe BE, Plotnikoff RC, Dewar DL, Collins CE, Lubans DR. Social cognitive mediators of dietary behavior change in adolescent girls. *American Journal of Health Behavior.* 2015;39(1):51-61. | No UK specific results |
| McGowan L, Pot GK, Stephen AM, Lavelle F, Spence M, Raats M, Hollywood L, McDowell D, McCloat A, Mooney E, Caraher M. The influence of socio-demographic, psychological and knowledge-related variables alongside perceived cooking and food skills abilities in the prediction of diet quality in adults: A nationally representative cross-sectional study. International *Journal of Behavioral Nutrition and Physical Activity*. 2016;13:1-3. | No UK specific results |
| McGrath AJ, Woodside JV, Wydenbach J. Children's eating behaviours and attitudes towards food in a deprived area of Belfast–a school-based survey. *Proceedings of the Nutrition Society.* 2012;71(OCE2):E82. | Abstract only |
| Mendonça N, Hill TR, Granic A, Davies K, Collerton J, Mathers JC, Siervo M, Wrieden WL, Seal CJ, Kirkwood TB, Jagger C. Macronutrient intake and food sources in the very old: analysis of the Newcastle 85+ Study. *British Journal of Nutrition*. 2016 Jun;115(12):2170-80. | Barriers or facilitators not assessed |
| Miguet M, Beaulieu K, Fillon A, Khammassi M, Masurier J, Lambert C, Duclos M, Boirie Y, Finlayson G, Thivel D. Effect of a 10-month residential multidisciplinary weight loss intervention on food reward in adolescents with obesity. *Physiology & Behavior.* 2020;223:112996. | No results for disadvantaged groups |
| Miller R, Spiro A, Stanner S. Micronutrient status and intake in the UK–where might we be in 10 years' time?. *Nutrition Bulletin*. 2016;41(1):14-41. | Does not link variables of interest |
| Millett C, Laverty AA, Stylianou N, Bibbins-Domingo K, Pape UJ. Impacts of a national strategy to reduce population salt intake in England: serial cross sectional study. *PLoS One*. 2012;7(1):e29836. | No adequate SES measure |
| Mills S, Adams J, Wrieden W, White M, Brown H. Sociodemographic characteristics and frequency of consuming home-cooked meals and meals from out-of-home sources: cross-sectional analysis of a population-based cohort study. *Public Health Nutrition*. 2018;21(12):2255-66. | Does not link variables of interest |
| Mills S, Wright T. Access to food retail outlets in County Durham, UK: a pragmatic cross-sectional study. *The Lancet*. 2015 ;385:S70. | Abstract only |
| Mizdrak A, Waterlander WE, Rayner M, Scarborough P. Using a UK virtual supermarket to examine purchasing behavior across different income groups in the United Kingdom: development and feasibility study. *Journal of Medical Internet Research*. 2017;19(10):e343. | Does not link variables of interest |
| Moffatt S, Lawson S, Patterson R, Holding E, Dennison A, Sowden S, Brown J. A qualitative study of the impact of the UK ‘bedroom tax’. *Journal of Public Health*. 2016;38(2):197-205. | No focus on healthy dietary intake |
| Molaodi OR, Leyland AH, Ellaway A, Kearns A, Harding S. Neighbourhood food and physical activity environments in England, UK: does ethnic density matter?. *International Journal of Behavioral Nutrition and Physical Activity*. 2012;9:1-1. | No focus on healthy dietary intake |
| Morris MA, Hulme C, Clarke GP, Edwards KL, Cade JE. What is the cost of a healthy diet? Using diet data from the UK Women's Cohort Study. *Journal of Epidemiology and Community Health*. 2014;68(11):1043-9. | Does not link variables of interest |
| Morris TT, Northstone K. Rurality and dietary patterns: associations in a UK cohort study of 10-year-old children. *Public Health Nutrition*. 2015;18(8):1436-43. | No results for disadvantaged groups |
| Mouratidou T, Ford FA, Fraser RB. Reproducibility and validity of a food frequency questionnaire in assessing dietary intakes of low‐income Caucasian postpartum women living in Sheffield, United Kingdom. *Maternal & Child Nutrition*. 2011;7(2):128-39. | Barriers or facilitators not assessed |
| Mullaney L, O'Higgins AC, Cawley S, Doolan A, McCartney D, Turner MJ. An estimation of periconceptional under-reporting of dietary energy intake*. Journal of Public Health*. 2015;37(4):728-36. | No focus on healthy dietary intake |
| Murphy M, Boardman F, Robertson W, Johnson R. Children's perspectives and experiences of health, diet, physical activity and weight in an urban, multi‐ethnic UK population: A qualitative study. *Child: Care, Health and Development*. 2021;47(5):597-607. | Barriers or facilitators not assessed |
| Naughton F, Ward E, Khondoker M, Belderson P, Marie Minihane A, Dainty J, Hanson S, Holland R, Brown T, Notley C. Health behaviour change during the UK COVID‐19 lockdown: Findings from the first wave of the C‐19 health behaviour and well‐being daily tracker study. *British Journal of Health Psychology*. 2021;26(2):624-43. | Does not link variables of interest |
| Naughton P, McCarthy M, McCarthy S. Reducing consumption of confectionery foods: A post-hoc segmentation analysis using a social cognition approach. *Appetite*. 2017;117:168-78. | No UK specific results |
| Nevill A, Donnelly P, Shibli S, Foster C, Murphy M. Modifiable behaviors help to explain the inequalities in perceived health associated with deprivation and social class: evidence from a national sample. *Journal of Physical Activity and Health*. 2014;11(2):339-47. | Barriers or facilitators not assessed |
| Newton JN, Briggs AD, Murray CJ, Dicker D, Foreman KJ, Wang H, Naghavi M, Forouzanfar MH, Ohno SL, Barber RM, Vos T. Changes in health in England, with analysis by English regions and areas of deprivation, 1990–2013: a systematic analysis for the Global Burden of Disease Study 2013. *The Lancet*. 2015;386(10010):2257-74. | Barriers or facilitators not assessed |
| Northstone K, Smith AD, Cribb VL, Emmett PM. Dietary patterns in UK adolescents obtained from a dual-source FFQ and their associations with socio-economic position, nutrient intake and modes of eating. *Public Health Nutrition*. 2014;17(7):1476-85. | Does not link variables of interest |
| Ntouva A, Tsakos G, Watt RG. Sugars consumption in a low-income sample of British young people and adults. *British Dental Journal*. 2013;215(1):E2-. | Barriers or facilitators not assessed |
| O’Connor L, Brage S, Griffin SJ, Wareham NJ, Forouhi NG. The cross-sectional association between snacking behaviour and measures of adiposity: the Fenland Study, UK. *British Journal of Nutrition*. 2015;114(8):1286-93. | No results for disadvantaged groups |
| Ochieng BM. Healthy weight maintenance strategy in early childhood: The views of black African migrant parents and health visitors. *Health & Social Care in the Community*. 2020;28(5):1551-9. | No results for disadvantaged groups |
| O'Connell R, Owen C, Padley M, Simon A, Brannen J. Which types of family are at risk of food poverty in the UK? A relative deprivation approach. *Social Policy and Society*. 2019;18(1):1-8. | Barriers or facilitators not assessed |
| Ohly H, Crossland N, Dykes F, Lowe N, Hall-Moran V. A realist review to explore how low-income pregnant women use food vouchers from the UK’s Healthy Start programme. *BMJ Open*. 2017;7(4):e013731. | Review combines results from UK and US studies |
| Papier K, Tong TY, Appleby PN, Bradbury KE, Fensom GK, Knuppel A, Perez-Cornago A, Schmidt JA, Travis RC, Key TJ. Comparison of major protein-source foods and other food groups in meat-eaters and non-meat-eaters in the EPIC-Oxford cohort. *Nutrients.* 2019;11(4):824. | Barriers or facilitators not assessed |
| Parnham J, Millett C, Chang K, von Hinke S, Pearson-Stuttard J, Vamos EP. The Healthy Start scheme and its association with food expenditure in low-income families in the UK. *European Journal of Public Health*. 2020;30(Supplement_5):ckaa166-193. | Abstract only |
| Parsons JM. Making time for food when ‘doing time’; how enhanced status prisoners counter the indignity of prison foodways. *Appetite*. 2020;146:104507. | No results for disadvantaged groups |
| Parsons JM. When convenience is inconvenient:‘Healthy’family foodways and the persistent intersectionalities of gender and class*. Journal of Gender Studies*. 2016;25(4):382-97. | No results for disadvantaged groups |
| Patel L, Alicandro G, La Vecchia C. Dietary approaches to stop hypertension (DASH) diet and associated socio-economic inequalities in the UK. *British Journal of Nutrition*. 2020 Nov;124(10):1076-85. | Barriers or facilitators not assessed |
| Pearce M, Bray I, Horswell M. Weight gain in mid-childhood and its relationship with the fast food environment*. Journal of Public Health.* 2018;40(2):237-44. | No focus on healthy dietary intake |
| Pearce MS, Relton CL, Groom A, Peaston RT, Francis RM. A lifecourse study of bone resorption in men ages 49–51 years: The Newcastle Thousand Families cohort study. *BONE*. 2010;46(4):952-6. | No focus on healthy dietary intake |
| Pechey R, Jebb SA, Kelly MP, Almiron-Roig E, Conde S, Nakamura R, Shemilt I, Suhrcke M, Marteau TM. Socioeconomic differences in purchases of more vs. less healthy foods and beverages: analysis of over 25,000 British households in 2010. *Social Science & Medicine*. 2013;92:22-6. | Barriers or facilitators not assessed |
| Pechey R, Monsivais P. Socioeconomic inequalities in the healthiness of food choices: Exploring the contributions of food expenditures. *Preventive Medicine*. 2016;88:203-9. | No adequate SES measure |
| Pechey R, Monsivais P. Supermarket choice, shopping behavior, socioeconomic status, and food purchases. *American Journal of Preventive Medicine*. 2015;49(6):868-77. | No adequate SES measure |
| Pelham-Burn SE, Frost CJ, Russell JM, Barker ME. Improving the nutritional quality of charitable meals for homeless and vulnerable adults. A case study of food provision by a food aid organisation in the UK. *Appetite*. 2014;82:131-7. | No focus on healthy dietary intake |
| Penn L, Dombrowski SU, Sniehotta FF, White M. Perspectives of UK Pakistani women on their behaviour change to prevent type 2 diabetes: qualitative study using the theory domain framework. *BMJ Open*. 2014;4(7):e004530. | No results for disadvantaged groups |
| Pettinger C, Parsons JM, Cunningham M, Withers L, D’Aprano G, Letherby G, Sutton C, Whiteford A, Ayres R. Engaging homeless individuals in discussion about their food experiences to optimise wellbeing: A pilot study. *Health Education Journal.* 2017;76(5):557-68. | No focus on healthy dietary intake |
| Phillips G, Renton A, Moore DG, Bottomley C, Schmidt E, Lais S, Yu G, Wall M, Tobi P, Frostick C, Clow A. The Well London program-a cluster randomized trial of community engagement for improving health behaviors and mental wellbeing: baseline survey results. *Trials.* 2012;13:1-5. | Barriers or facilitators not assessed |
| Pinho-Gomes AC, Knight A, Critchley J, Pennington M. Addressing the low consumption of fruit and vegetables in England: a cost-effectiveness analysis of public policies. *Journal of Epidemiology and Community Health*. 2021;75(3):282-8. | No focus on healthy dietary intake |
| Porter J, Ravaghi V, Hill KB, Watt RG. Oral health behaviours of children in England, Wales and Northern Ireland 2013. *British Dental Journal*. 2016 Sep;221(5):263-8. | No focus on healthy dietary intake (majority of participants under 18) |
| Poulter H, Eberhardt J, Moore H, Windgassen S. '‘Absorbing the shock’: Food scarcity and eating behaviours within the context of in-work poverty in North East England. *European and International Congress on Obesity*, 2020 Sep 1. | Abstract only |
| Preston H, Burley VJ. What's in a food bag? Analysis of the content of food bags provided by the Bradford Metropolitan Food Bank. *Proceedings of the Nutrition Society.* 2015;74(OCE1):E136. | Barriers or facilitators not assessed |
| Pringle A, Zwolinsky S, McKenna J, Robertson S, Daly-Smith A, White A. Health improvement for men and hard-to-engage-men delivered in English Premier League football clubs. *Health Education Research*. 2014;29(3):503-20. | No results for disadvantaged groups |
| Purdam, K., Esmail, A. and Garratt, E., 2019. Food insecurity amongst older people in the UK. *British Food Journal*, 121(3), pp.658-674. | No focus on healthy dietary intake |
| Ramsay SE, Arianayagam DS, Papacosta O, Lennon LT, Wannamethee SG. Cardiometabolic and social determinants of frailty: results from a population-based study of elderly British men. *Age and Ageing*. 2014;43(suppl_2):ii16-. | Barriers or facilitators not assessed |
| Rawlins E, Baker G, Maynard M, Harding S. Perceptions of healthy eating and physical activity in an ethnically diverse sample of young children and their parents: the DEAL prevention of obesity study. *Journal of Human Nutrition and Dietetics*. 2013;26(2):132-44. | Does not link variables of interest |
| Reeves S, Halsey LG, McMeel Y, Huber JW. Breakfast habits, beliefs and measures of health and wellbeing in a nationally representative UK sample. *Appetite*. 2013;60:51-7. | Does not link variables of interest |
| Reidlinger DP, Sanders TA, Goff LM. How expensive is a cardioprotective diet? Analysis from the CRESSIDA study. *Public Health Nutrition*. 2017;20(8):1423-30. | No focus on healthy dietary intake |
| Relton C, Li J, Strong M, Holdsworth M, Cooper R, Green M, Bissell P. Deprivation, clubs and drugs: results of a UK regional population-based cross-sectional study of weight management strategies. *BMC Public Health*. 2014;14:1-1. | No focus on healthy dietary intake |
| Richards J, Kliner M, Brierley S, Stroud L. Maternal and infant health of Eastern Europeans in Bradford, UK: a qualitative study. *Community Practice*. 2014;87(9):33-6. | No focus on healthy dietary intake |
| Rippin HL, Hutchinson J, Ocke M, Jewell J, Breda JJ, Cade JE. An exploration of socio-economic and food characteristics of high trans fatty acid consumers in the Dutch and UK national surveys after voluntary product reformulation. *Food & Nutrition Research*. 2017. | Does not link variables of interest |
| Robertson J, Emerson E, Baines S, Hatton C. Obesity and health behaviours of British adults with self-reported intellectual impairments: cross sectional survey. *BMC Public Health*. 2014;14:1-7. | No results for disadvantaged groups |
| Robertson T, Benzeval M, Whitley E, Popham F. The role of material, psychosocial and behavioral factors in mediating the association between socioeconomic position and allostatic load (measured by cardiovascular, metabolic and inflammatory markers). *Brain, Behavior, and Immunity*. 2015;45:41-9. | No focus on healthy dietary intake |
| Robertson W, Fleming J, Kamal A, Hamborg T, Khan KA, Griffiths F, Stewart-Brown S, Stallard N, Petrou S, Simkiss D, Harrison E. Randomised controlled trial and economic evaluation of the ‘Families for Health’programme to reduce obesity in children. *Archives of Disease in Childhood.* 2017;102(5):416-26. | No results for disadvantaged groups |
| Robinson M, Robertson S, McCullagh J, Hacking S. Working towards men’s health: Findings from the Sefton men’s health project. *Health Education Journal*. 2010;69(2):139-49. | No focus on healthy dietary intake |
| Rogers S, Pryer JA. Who consumed 5 or more portions of fruit and vegetables per day in 1986–1987 and in 2000–2001?. *Public Health Nutrition*. 2012;15(7):1240-7. | Does not link variables of interest |
| Rose AK, Hardman CA, Christiansen P. The effects of a priming dose of alcohol and drinking environment on snack food intake*. Appetite*. 2015;95:341-8. | No results for disadvantaged groups |
| Sarkar C, Webster C, Gallacher J. Are exposures to ready-to-eat food environments associated with type 2 diabetes? A cross-sectional study of 347 551 UK Biobank adult participants*. The Lancet Planetary Health*. 2018;2(10):e438-50. | No focus on healthy dietary intake |
| Saunders P, Saunders A, Middleton J. Living in a ‘fat swamp’: exposure to multiple sources of accessible, cheap, energy-dense fast foods in a deprived community. *British Journal of Nutrition*. 2015;113(11):1828-34. | No focus on healthy dietary intake |
| Sauveplane-Stirling V, Crichton D, Tessier S, Parrett A, Garcia AL. The food retail environment and its use in a deprived, urban area of Scotland. *Public Health*. 2014;128(4):360-6. | Does not link variables of interest |
| Schalkwijk AA, van der Zwaard BC, Nijpels G, Elders PJ, Platt L. The impact of greenspace and condition of the neighbourhood on child overweight. *The European Journal of Public Health.* 2018;28(1):88-94. | No focus on healthy dietary intake |
| Scheelbeek PF, Cornelsen L, Marteau TM, Jebb SA, Smith RD. Potential impact on prevalence of obesity in the UK of a 20% price increase in high sugar snacks: modelling study. *BMJ*. 2019;366. | No focus on healthy dietary intake |
| Shaw H. Food access, diet and health in the UK: an empirical study of Birmingham. *British Food Journal*. 2012;114(4):598-616. | Does not link variables of interest |
| Shinwell J, Defeyter MA. Food insecurity: a constant factor in the lives of low-income families in Scotland and England. *Frontiers in Public Health*. 2021 Ma;9:588254. | No focus on healthy dietary intake |
| Smith NR, Kelly YJ, Nazroo JY. The effects of acculturation on obesity rates in ethnic minorities in England: evidence from the Health Survey for England. The European Journal of Public Health. 2012;22(4):508-13. | Barriers or facilitators not assessed |
| Soltani N. Dietary practices of Iranian migrant women in the United Kingdom. *Appetite*. 2021;162:105144. | No results for disadvantaged groups |
| Spence S, Matthews JN, McSweeney L, Rowland M, Orango P, Adamson AJ. A natural experimental evaluation of the effect of universal infant free school meals on key stage 1 pupil's dietary intake in northeast England: a pilot study. *The Lancet*. 2019;394:S87. | Abstract only |
| Spyreli E, McKinley MC, Woodside JV, Kelly C. Using photo-elicitation to explore the impact of COVID-19 restrictions on food decisions of low-income families in Northern Ireland. *Proceedings of the Nutrition Society*. 2021 Jan;80(OCE3):E82. | Abstract only |
| Stait E, Calnan M. Are differential consumption patterns in health-related behaviours an explanation for persistent and widening social inequalities in health in England?. *International Journal for Equity in Health*. 2016;15:1-1. | Does not link variables of interest |
| Standage M, Cumming SP, Gillison FB. A cluster randomized controlled trial of the be the best you can be intervention: effects on the psychological and physical well-being of school children*. BMC Public Health*. 2013;13:1-0. | No results for disadvantaged groups |
| Stevens R, Kelaiditi E, Myrissa K. Exploration of the dietary habits, lifestyle patterns and barriers to healthy eating in UK post‐partum women*. Nutrition Bulletin*. 2021;46(1):26-39. | No results for disadvantaged groups |
| Stringhini S, Dugravot A, Shipley M, Goldberg M, Zins M, Kivimäki M, Marmot M, Sabia S, Singh-Manoux A. Health behaviours, socioeconomic status, and mortality: further analyses of the British Whitehall II and the French GAZEL prospective cohorts. *PLoS Medicine*. 2011;8(2):e1000419. | Barriers or facilitators not assessed |
| Stringhini S, Sabia S, Shipley M, Brunner E, Nabi H, Kivimaki M, Singh-Manoux A. Association of socioeconomic position with health behaviors and mortality. *JAMA.* 2010;303(12):1159-66. | Barriers or facilitators not assessed |
| Strömmer S, Weller S, Morrison L, Soltani H, Stephenson J, Whitworth M, Rundle R, Brewin J, Poston L, Lawrence W, Barker M. Young women's and midwives' perspectives on improving nutritional support in pregnancy: The babies, eating, and LifestyLe in adolescence (BELLA) study. *Social Science & Medicine*. 2021;274:113781. | No results for disadvantaged groups |
| Taher AK, Evans N, Evans CE. The cross-sectional relationships between consumption of takeaway food, eating meals outside the home and diet quality in British adolescents. *Public Health Nutrition*. 2019;22(1):63-73. | Majority of participants under 18 |
| Thomas F, Thomas C, Hooper L, Rosenberg G, Vohra J, Bauld L. Area deprivation, screen time and consumption of food and drink high in fat salt and sugar (HFSS) in young people: results from a cross-sectional study in the UK. *BMJ Open*. 2019 ;9(6):e027333. | Majority of participants under 18 |
| Thompson C, Ponsford R, Lewis D, Cummins S. Fast-food, everyday life and health: A qualitative study of ‘chicken shops’ in East London. *Appetite.* 2018;128:7-13. | No focus on healthy dietary intake |
| Thompson JL, Bentley G, Davis M, Coulson J, Stathi A, Fox KR. Food shopping habits, physical activity and health-related indicators among adults aged≥ 70 years. *Public Health Nutrition*. 2011 Sep;14(9):1640-9. | Does not link variables of interest |
| Thompson JL, Bentley G, Davis M, Coulson J, Stathi A, Fox KR. Food shopping habits, physical activity and health-related indicators among adults aged≥ 70 years. *Public Health Nutrition*. 2011;14(9):1640-9. | Does not link variables of interest |
| Tiffin R, Arnoult M. The demand for a healthy diet: estimating the almost ideal demand system with infrequency of purchase. *European Review of Agricultural Economics*. 2010;37(4):501-21. | Barriers or facilitators not assessed |
| Timmins KA, Hulme C, Cade JE. The monetary value of diets consumed by British adults: an exploration into sociodemographic differences in individual-level diet costs. *Public Health Nutrition*. 2015;18(1):151-9. | No focus on healthy dietary intake |
| Toumpakari Z, Tilling K, Haase AM, Johnson L. High-risk environments for eating foods surplus to requirements: a multilevel analysis of adolescents’ non-core food intake in the National Diet and Nutrition Survey (NDNS). *Public Health Nutrition*. 2019;22(1):74-84. | Majority of participants under 18 |
| Tully L, Allen‐Walker V, Spyreli E, McHugh S, Woodside JV, Kearney PM, McKinley MC, Dean M, Kelly C. Solid advice: complementary feeding experiences among disadvantaged parents in two countries. *Maternal & Child Nutrition*. 2019;15(3):e12801. | No UK specific results |
| Tyrrell J, Wood AR, Ames RM, Yaghootkar H, Beaumont RN, Jones SE, Tuke MA, Ruth KS, Freathy RM, Davey Smith G, Joost S. Gene–obesogenic environment interactions in the UK Biobank study. *International Journal of Epidemiology*. 2017;46(2):559-75. | Does not link variables of interest |
| van den Heuvel E, Murphy JL, Appleton KM. The provision of recipes and single-use herb/spice packets to increase egg and protein intake in community-dwelling older adults: a randomised controlled trial. *Public Health Nutrition*. 2021;24(8):2109-22. | No results for disadvantaged groups |
| Van Kesteren R, Evans A. Cooking without thinking: How understanding cooking as a practice can shed new light on inequalities in healthy eating. *Appetite*. 2020;147:104503. | No focus on healthy dietary intake |
| Vávra J, Megyesi B, Duží B, Craig T, Klufová R, Lapka M, Cudlínová E. Food self‐provisioning in Europe: an exploration of sociodemographic factors in five regions. *Rural Sociology.* 2018;83(2):431-61. | Barriers or facilitators not assessed |
| Vincent S, Jopling M. The health and well‐being of children and young people who are looked after: Findings from a face‐to‐face survey in Glasgow. *Health & Social Care in the Community*. 2018;26(2):182-90. | Majority of participants under 18 |
| Vogel C, Lewis D, Ntani G, Cummins S, Cooper C, Moon G, Baird J. The relationship between dietary quality and the local food environment differs according to level of educational attainment: A cross-sectional study. *PloS Oone.* 2017;12(8):e0183700. | No results for disadvantaged groups |
| Ward M, Berry DJ, Power C, Hyppönen E. Working patterns and vitamin D status in mid-life: a cross-sectional study of the 1958 British birth cohort. *Occupational and Environmental Medicine.* 2011;68(12):902-7. | No focus on healthy dietary intake |
| Watt RG, Draper AK, Ohly HR, Rees G, Pikhart H, Cooke L, Moore L, Crawley H, Pettinger C, McGlone P, Hayter AK. Methodological development of an exploratory randomised controlled trial of an early years' nutrition intervention: the CHERRY programme (Choosing Healthy Eating when Really Young). *Maternal & Child Nutrition*. 2014;10(2):280-94. | No focus on healthy dietary intake |
| Webster C. Relationship of total 25-OH vitamin D concentrations to Indices of Multiple Deprivation: geoanalysis of laboratory results. *Annals of Clinical Biochemistry*. 2013;50(1):31-8. | Barriers or facilitators not assessed |
| Whitehead R, Currie D, Inchley J, Currie C. Educational expectations and adolescent health behaviour: an evolutionary approach. International *Journal of Public Health*. 2015;60:599-608. | Does not link variables of interest |
| Whitley E, Batty GD, Hunt K, Popham F, Benzeval M. The role of health behaviours across the life course in the socioeconomic patterning of all-cause mortality: the west of Scotland twenty-07 prospective cohort study. *Annals of Behavioral Medicine*. 2014;47(2):148-57. | No focus on healthy dietary intake |
| Whybrow S, Craig LC, Macdiarmid JI. Dietary patterns of households in Scotland: Differences by level of deprivation and associations with dietary goals. *Nutrition and Health*. 2018;24(1):29-35. | Barriers or facilitators not assessed |
| Whybrow S, Hollis JL, Macdiarmid JI. Social deprivation is associated with poorer adherence to healthy eating dietary goals: analysis of household food purchases*. Journal of Public Health.* 2018;40(1):e8-15. | Barriers or facilitators not assessed |
| Whybrow S, Horgan GW, Macdiarmid JI. Buying less and wasting less food. Changes in household food energy purchases, energy intakes and energy density between 2007 and 2012 with and without adjustment for food waste. *Public Health Nutrition*. 2017;20(7):1248-56. | Barriers or facilitators not assessed |
| Wildman JM, Valtorta N, Moffatt S, Hanratty B. ‘What works here doesn’t work there’: The significance of local context for a sustainable and replicable asset‐based community intervention aimed at promoting social interaction in later life. *Health & Social Care in the Community.* 2019;27(4):1102-10. | No focus on healthy dietary intake |
| Wills W, Backett-Milburn K, Roberts ML, Lawton J. The framing of social class distinctions through family food and eating practices. *The Sociological Review*. 2011;59(4):725-40. | Does not link variables of interest |
| Zhang YB, Chen C, Pan XF, Guo J, Li Y, Franco OH, Liu G, Pan A. Associations of healthy lifestyle and socioeconomic status with mortality and incident cardiovascular disease: two prospective cohort studies. *BMJ.* 2021;373. | Barriers or facilitators not assessed |
| Ziauddeen N, Almiron-Roig E, Penney TL, Nicholson S, Kirk SF, Page P. Eating at food outlets and “on the go” is associated with less healthy food choices in adults: cross-sectional data from the UK National Diet and Nutrition Survey Rolling Programme (2008–2014). *Nutrients.* 2017;9(12):1315. | Barriers or facilitators not assessed |
